# Relevance, redundancy, and complementarity trade-off (RRCT): A principled, generic, robust feature-selection tool

**DOI:** 10.1016/j.patter.2022.100471

**Published:** 2022-03-31

**Authors:** Athanasios Tsanas

**Affiliations:** 1 Usher Institute, Edinburgh Medical School, University of Edinburgh, NINE Edinburgh BioQuarter, 9 Little France road, Edinburgh, UK; 2 School of Mathematics, University of Edinburgh, Edinburgh, UK; 3 Alan Turing Institute, British Library, London, UK

**Keywords:** curse of dimensionality, dimensionality reduction, feature selection, statistical learning, principle of parsimony, variable selection, information theory

## Abstract

We present a new heuristic feature-selection (FS) algorithm that integrates in a principled algorithmic framework the three key FS components: relevance, redundancy, and complementarity. Thus, we call it relevance, redundancy, and complementarity trade-off (RRCT). The association strength between each feature and the response and between feature pairs is quantified via an information theoretic transformation of rank correlation coefficients, and the feature complementarity is quantified using partial correlation coefficients. We empirically benchmark the performance of RRCT against 19 FS algorithms across four synthetic and eight real-world datasets in indicative challenging settings evaluating the following: (1) matching the true feature set and (2) out-of-sample performance in binary and multi-class classification problems when presenting selected features into a random forest. RRCT is very competitive in both tasks, and we tentatively make suggestions on the generalizability and application of the best-performing FS algorithms across settings where they may operate effectively.

## Highlights

• New feature-selection algorithm, RRCT, is robust and computationally efficient
• RRCT is parameter-free and deployable off-the-shelf
• Applicable in datasets with mixed-type variables, regression, and classification
• Thorough empirical evaluation: 20 feature-selection algorithms, 12 diverse datasets

## The bigger picture

High-dimensional datasets are now increasingly met across a wide span of data-science applications, where the large number of variables may obstruct the extraction of useful patterns in the data and often prove detrimental to the subsequent supervised learning process (regression or classification). The problem is known as the curse of dimensionality and is often tackled using feature-selection algorithms. This study proposes an accurate, robust, computationally efficient feature-selection algorithm that is applicable in mixed-type variable settings across both regression and classification while inherently accounting for all key properties in determining a parsimonious feature subset: relevance, redundancy, and conditional relevance (or complementarity). Selecting a robust feature subset can save on data-collection resources, reduce computational cost and statistical-model portability, enhance interpretability, and often increase model generalization performance.

## Graphical abstract

**Figure:**
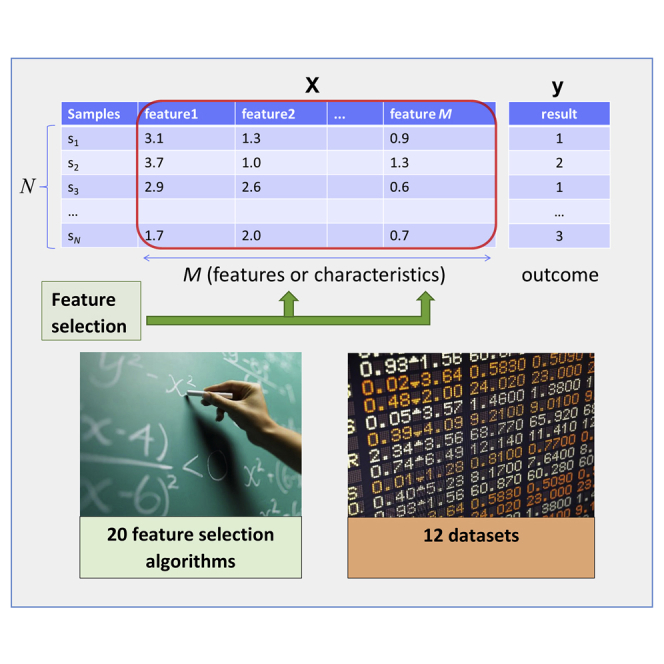

## Teaser

Many practical datasets comprise a large number of variables, a setting that poses considerable challenges in terms of gaining insights into that particular domain. In this study, we introduce a robust algorithm that can identify a subset of the original variables, which should be retained for further analysis, and demonstrate its performance side by side against 19 state-of-art competing algorithms across 12 diverse datasets. Our findings have important implications toward reducing data-collection resources and interpretability as well as improving potential insights into the studied problem.

## Introduction

There has been continuously growing research and commercial interest in collecting and processing data across diverse applications, ranging from healthcare to finance, military, and others. Typically, in most data-science applications, we want to infer the statistical and functional relationship between a set of features (characteristics of the dataset) and a (measured or assessed) quantity of interest known as the response (or outcome); this is commonly referred to as the supervised learning setup.^1^ Increasingly, datasets are becoming more complex, often having an abundance of (recorded or extracted) features. The presence of a large number of features often obstructs the interpretation of useful patterns in the data and may be detrimental to the subsequent learning process of mapping features to the response.^2^, ^3^, ^4^ This problem, widely known as the curse of dimensionality,^1^ occurs because the feature space is sparsely populated: the number of required data samples to adequately populate the feature space grows exponentially with the number of features. This is further exacerbated in applications where the number of features is considerably larger than the number of data samples (also known as fat datasets, e.g., in micro-array data analysis problems).^1^

To mitigate the practical challenges arising because of the curse of dimensionality, researchers often employ feature-transformation or feature-selection (FS) methods. Feature transformation aims to build a new feature space of reduced dimensionality, producing a compact representation of the information that may be distributed across several of the original features. Although it has shown promising results in different applications,^3^^,^^5^^,^^6^ feature transformation is usually not easily interpretable because the physical meaning of the original features cannot be retrieved. In addition, it does not save on resources required during the data-collection process since all original features still need to be measured or computed. Moreover, in very high dimensional settings where the number of irrelevant features may exceed the number of relevant features, reliable feature transformation can be challenging.^5^

FS algorithms abound in the literature, and there has been continued research interest in their development^2^^,^^3^^,^^7^, ^8^, ^9^, ^10^, ^11^, ^12^, ^13^, ^14^ and exploration to gain application-specific insights.^3^^,^^15^, ^16^, ^17^, ^18^ The FS algorithms reduce the original (high-dimensional) feature set onto a feature subset by discarding features aiming to (1) reduce computational time (depending on the application, also save on data-collection resources), (2) improve prediction performance in a standard supervised learning setup, and (3) provide new insights into the studied application (focusing on specific features of interest). FS algorithms can be broadly grouped into three categories: (1) filters, (2) embedded methods, and (3) wrappers. Wrappers and embedded methods incorporate a statistical learner (classifier or regressor), whereas filters are independent of the statistical learner. Wrappers incorporate a statistical learner and search the feature subset space using the performance of the statistical learner (formally assessed through the chosen loss function and using training and testing subsets). Embedded methods determine the feature subsets that best contribute to the performance of the statistical learning model while building the model itself (this is formalized through the loss function).^3^^,^^5^^,^^19^ Arguably, wrappers and embedded methods for FS have the following shortcomings compared with filters: (1) they often (but not always) have greater computational complexity, which is exacerbated as the dataset grows larger, (2) the selected feature subset for a specific statistical learner may be sub-optimal for a different statistical learner, a problem known as feature exportability, (3) controlling internal parameters (parameter fine-tuning) of the statistical learner requires experimentation and expertise and is time-consuming, and (4) there are inherent statistical-learner constraints; for example, some do not handle multi-class classification or regression problems. The problem with feature exportability arises because the features chosen in a wrapper or embedded algorithm are tailored to optimize the performance of the specific statistical learner. Therefore, the selected feature subset may not reflect the global properties of the dataset and might not generalize well in alternative statistical learners.^20^ Filters attempt to overcome these limitations and commonly evaluate feature subsets based on their information content (for example, using statistical tests and statistical properties of the data) instead of optimizing the performance of specific statistical learners and are usually computationally more efficient. Henceforth, in this study, FS is used to refer exclusively to filters.

Historically, filter FS algorithms were developed to be computationally efficient using statistical properties of the data by applying statistical hypothesis tests and using correlation-based concepts to rank features and, progressively, have become considerably more sophisticated.^3^^,^^21^ Some of the filter FS algorithms are computationally very demanding (for example, relying on high-dimensional density estimates, computationally intensive optimization, or computing mutual information [MI], which is computationally expensive and practically challenging with reduced data samples) and some require careful fine-tuning of internal parameters to optimize performance, while others are limited in their application because they can only address binary classification problems or cannot be generalized to regression settings. For an overview of these challenges, we refer to Guyon et al.^3^ and Deng et al.^21^ Crucially, some studies highlight the importance of using simple filters before experimenting with more sophisticated schemes, remarking that many promising but elementary concepts have been left unexplored.^3^^,^^22^, ^23^, ^24^

Motivated by the last statement, we pursued the development of a generic, computationally efficient FS algorithm that would be applicable across almost any data-science problem which is presented in the form of a data matrix and a response so that it could serve as an off-the-shelf FS algorithm. This led to the development of the new correlation-based filter FS algorithm that we propose here, which we call relevance, redundancy, and complementarity trade-off (RRCT). RRCT uses a simple nonlinear transformation of the correlation coefficients using information theoretic concepts to quantify the association of the features with the response and the overlapping information between features and also explicitly takes into account feature interactions as an integral component toward FS.

The aims of this study are to (1) introduce and empirically validate the new RRCT FS algorithm across a range of diverse datasets and (2) provide an empirical comparison of various widely used filter-based FS algorithms across datasets (including fat datasets) to benchmark performance.

## Results

There are two approaches to assess the performance of FS algorithms. The first is by using a synthetic dataset where we know the ground truth (i.e., the features that are, by design, functionally associated with the response: these are known as the true features, and, correspondingly, the remaining features are known as false features or probes). The second approach is when we do not know the true features, e.g., in real-world datasets, and hence, we present the selected feature subsets into a statistical learner so that we infer FS performance on the basis of a chosen performance measure. For further details see FS assessment in the experimental procedures.

### Validating FS algorithms using synthetic data

**Figure 1:**
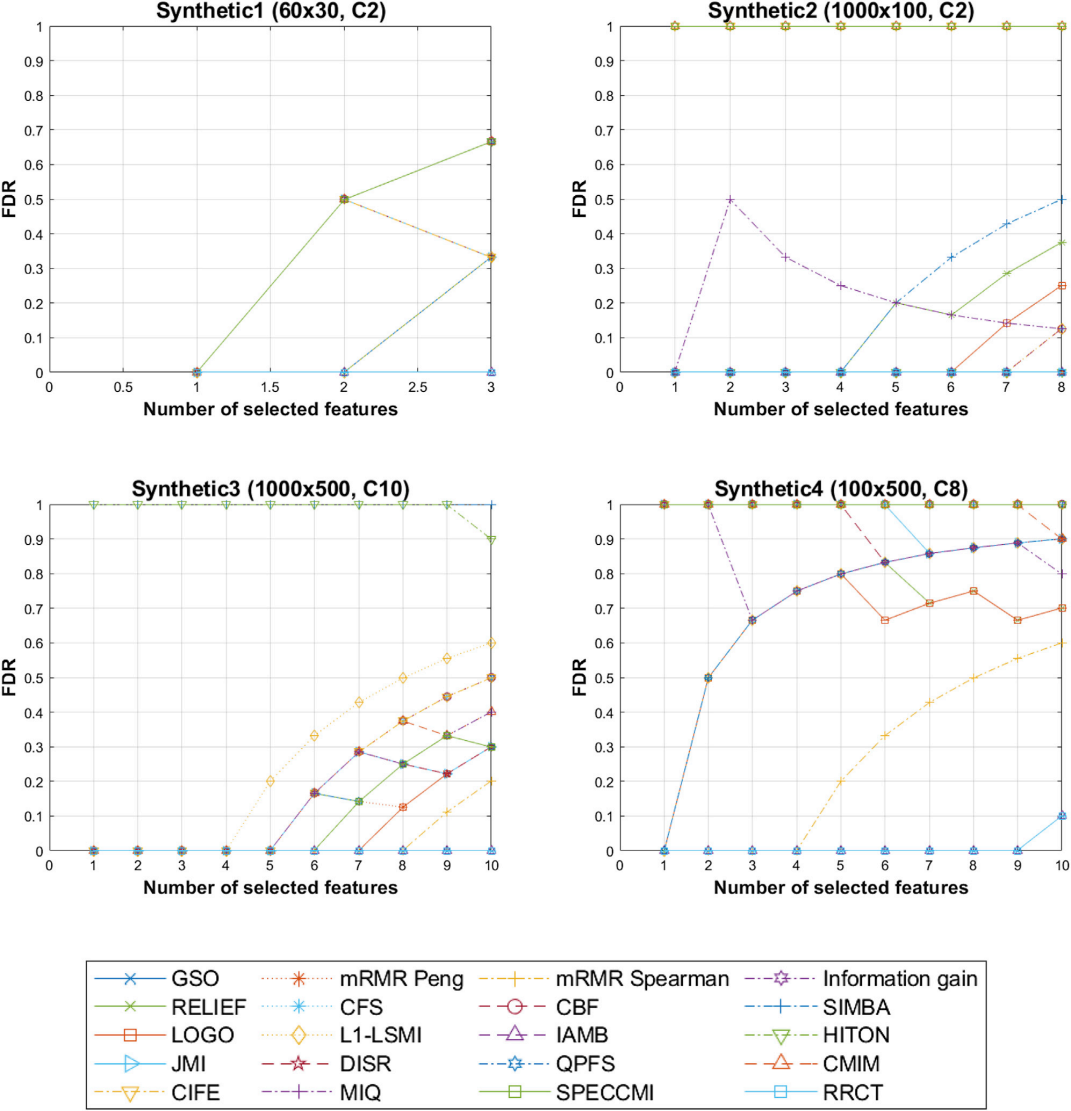
Comparison of the feature-selection algorithms in terms of true feature set recovery The lower the false discovery rate (FDR), the better the feature-selection algorithm. The horizontal axis denotes the number of features selected during the incremental process for the number of true features in each dataset (the first synthetic dataset had 3 true features, the second had 8 true features, the third and fourth had 10 true features). Above each plot in parentheses, we present the size of the design matrix (in the form samples × features) followed by the number of classes (e.g., the first dataset contains 60 samples and 30 features in a 2-class classification problem).

We begin the assessment of the 20 FS algorithms used in the study by reporting the false discovery rate (FDR) outputs for the four synthetic datasets (findings in Figure 1). These results should be interpreted sequentially: each step on the x axis denotes the iterative step in the FS algorithms, and the values in the y axis denote whether each FS algorithm’s choice identified true features in the subset or whether it selected a probe (false features, which are not members of the jointly minimal feature subset predicting the response). For example, a value of 1 in the y axis for the first iterative step for one of the FS algorithms would denote that the first feature that is selected for the given FS algorithm is a probe.

For convenience, Table 1 summarizes the FDR for the number of true features in each of the datasets and can be studied along with Figure 1. The results in Figure 1 and Table 1 illustrate that GSO, IAMB, and RRCT are all very effective at discarding probes. On the contrary, we remark that popular algorithms such as RELIEF, mRMR Peng (which is the default implementation most studies use when referring to mRMR), correlation-based FS (CFS), and JMI may stumble and erroneously select probes.

**Table 1:** False discovery rate for the synthetic datasets

	Synthetic 1 [60 × 30, C2]	Synthetic 2 [1,000 × 100, C2]	Synthetic 3 [1,000 × 500, C10]	Synthetic 4 [100 × 500, C8]
GSO	0	0	0	0.10
mRMR Peng	0.67	0	0.30	0.90
mRMR Spearman	0.33	0	0.20	0.60
Information gain	0.67	0	0.50	1
RELIEF	0.33	0.38	0.30	0.70
CFS	0.67	0.13	0.50	1
CBF	0.67	0.13	0.50	1
SIMBA	0.33	0.50	1	0.80
LOGO	0.67	0.25	0.30	0.70
L1-LSMI	0.33	0.13	0.60	0.90
IAMB	0	0	0	0.10
HITON	0.67	0	0.90	1
JMI	0.33	0	0.30	0.90
DISR	0.33	0	0.30	0.90
QPFS	0.67	0	0.30	0.90
CMIM	0.66	1	0.40	0.90
CIFE	0.33	1	0.50	1
MIQ	0.67	0.13	0.40	0.80
SPECCMI	0.67	1	0.30	1
RRCT	0	0	0	0.10

The design matrices are summarized in the form *N×M* [number of samples × number of features], and the following term indicates the problem and number of classes (e.g., C2 indicates that this is a classification problem with two classes). The presented results are the FDR scores for the number of true features in each of the datasets (see Figure 1 and also the description of the synthetic datasets for details).

Specifically, the first synthetic dataset (using Breiman’s generator) has been challenging for many FS algorithms due to the limited number of samples (n = 60); methods that require the computation of feature densities or the MI require a large number of samples to be able to robustly compute these quantities, and hence, their internal criteria toward selecting the feature set are likely compromised. This would explain why, for example, methods such as mRMR Peng, MIQ, and others made a mistaken selection of feature on the second step (see Figure 1). Similarly, this simple synthetic dataset has been challenging for other advanced FS methods including QPFS and SPECCMI, which cannot obtain an overall global MI-based assessment of the problem.

The second synthetic dataset should similarly be relatively easy given we have binary features and a straightforward computation of the response using 8 features. However, we note that many of the FS algorithms, notably SIMBA, SPECCMI, CMIM, and CIFE, do not perform well.

The third and fourth synthetic datasets are more challenging in the way they were generated, with features spanning across different orders of magnitude and exhibiting more complicated relationships associating the features with the response. The third synthetic dataset, for example, may be challenging for some algorithms due to the relatively large number of classes. Despite having a large number of samples (e.g., as opposed to the first synthetic dataset) and given that the features had been generated using normal distributions, again, most FS algorithms started selecting probes from the fourth and fifth steps (see Figure 1). In particular, the fourth dataset was generated to assess how well FS algorithms can recover features in a fat dataset, which is a well-known and challenging problem, and we see that none of the studied FS algorithms could recover all true features. Nevertheless, GSO, IAMB, and RRCT were very promising and only missed the last true feature on the 10^th^ step. All other FS algorithms investigated here appear to struggle to correctly discard probes.

Collectively, the experiments using synthetic datasets highlight some of the key weaknesses of FS algorithms across indicative types of data problems which could be seen in practical applications. We defer further elaboration on these findings for the Discussion.

### Validating FS algorithms using real-world data

**Figure 2:**
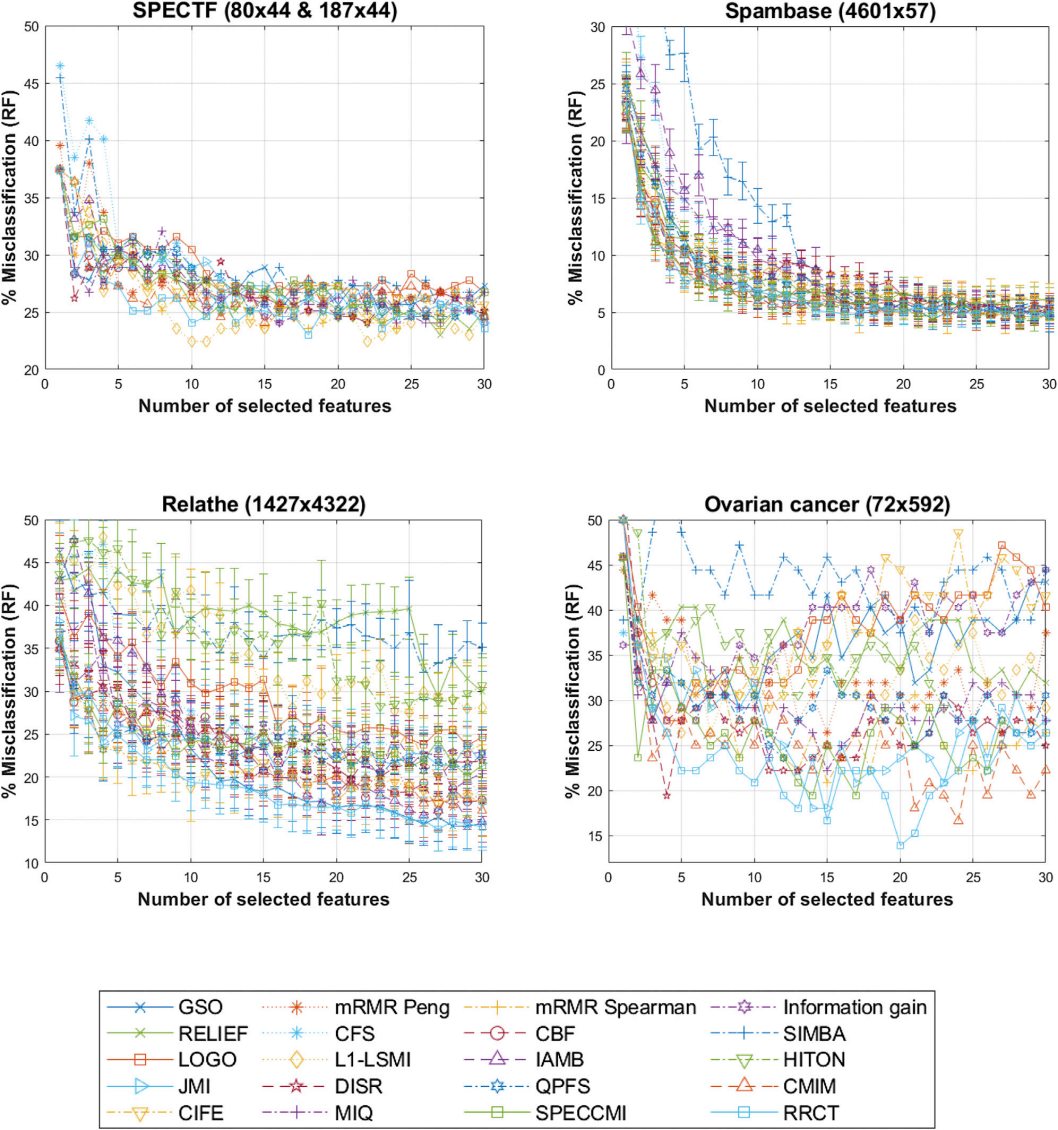
Comparison of the feature-selection algorithms based on RF out of sample performance for the binary-classification datasets The horizontal axis denotes the number of features selected in the greedy feature-selection process. When the assessment was done using 10-fold cross validation, the results are presented in the form mean ± SD (where SD is in the form of error bars).

**Figure 3:**
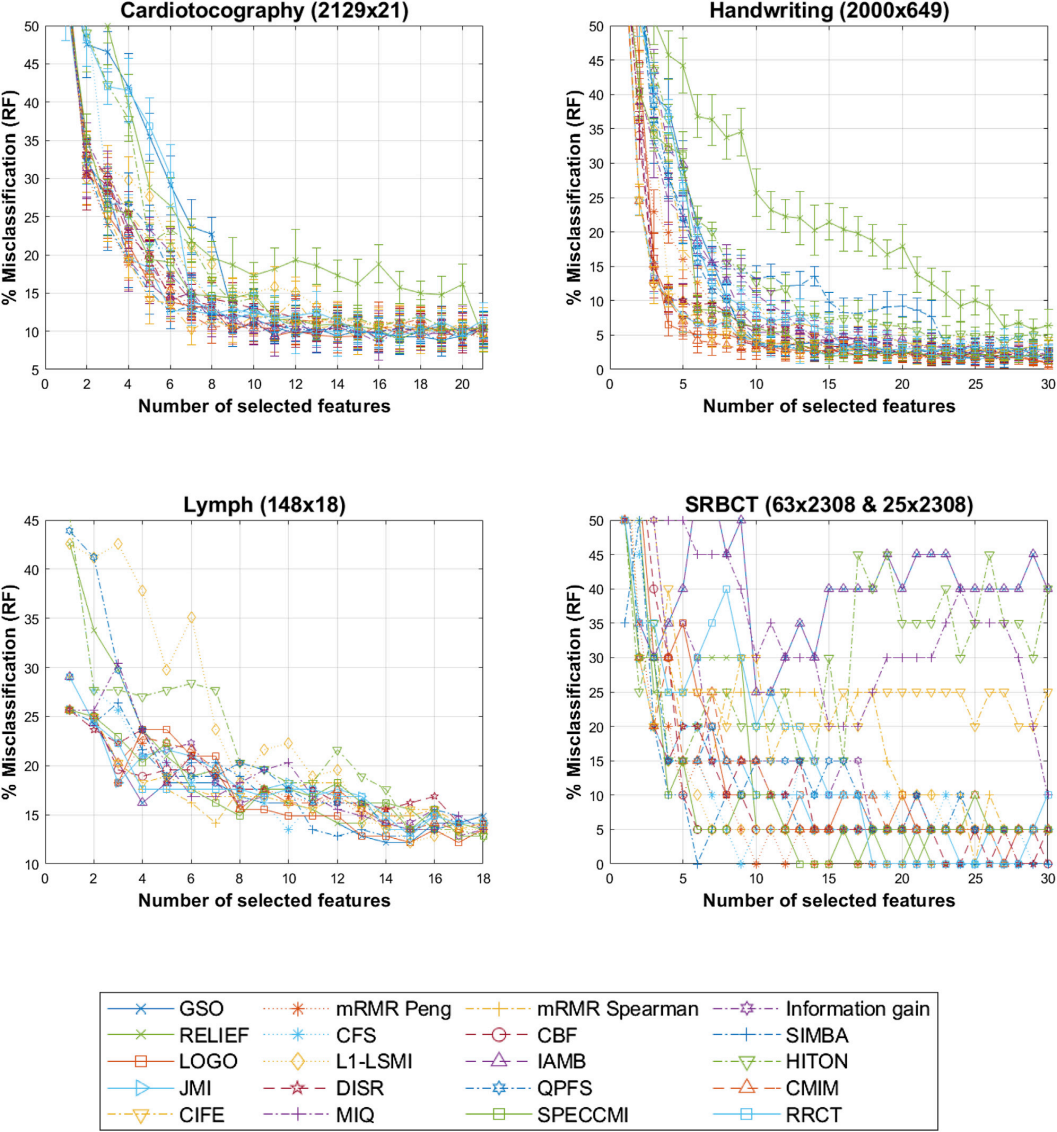
Comparison of the feature-selection algorithms based on RF out of sample performance for the multi-class classification datasets The horizontal axis denotes the number of features selected in the greedy feature-selection process. When the assessment was done using 10-fold cross validation, the results are presented in the form mean ± SD (where SD is in the form of error bars).

Figure 2 presents the results for binary classification settings, and Figure 3 presents the results for multi-class classification settings, as a function of the number of selected features presented into the random forest (RF). These results are summarized in Tables 2 (binary classification problems) and 3 (multi-class classification problems), highlighting the number of features that led to the lowest misclassification error for each FS algorithm. The overall impression here is that there is no clear winner among the competing FS algorithms in terms of performance, although we remark that the algorithm proposed in this study, RRCT, works very well generally.

**Table 2:** Out of sample RF, the percentage of misclassification for the binary-classification datasets

	SPECTF [80 × 44; 187 × 44]	Spambase [4,601 × 57]	Relathe [1,427 × 4,322]	Ovarian cancer [72 × 592]
GSO	24.60 (23)	4.37 ± 0.87 (22)	14.25 ± 2.66 (28)	27.78 (3)
mRMR Peng	24.60 (20)	4.39 ± 0.57 (29)	16.48 ± 3.26 (28)	26.39 (15)
mRMR Spearman	23.53 (18)	4.78 ± 1.67 (27)	20.14 ± 3.56 (27)	20.83 (15)
Information gain	24.06 (16)	4.65 ± 0.59 (29)	20.63 ± 3.93 (23)	27.78 (4)
RELIEF	22.99 (27)	5.61 ± 1.00 (24)	29.30 ± 3.99 (27)	29.17 (7)
CFS	25.13 (12)	5.85 ± 0.53 (17)	17.18 ± 2.33 (30)	29.17 (3)
CBF	28.34 (2)	5.07 ± 0.48 (18)	16.06 ± 2.30 (27)	31.94 (3)
SIMBA	25.13 (25)	4.89 ± 0.83 (25)	32.25 ± 3.23 (26)	37.50 (20)
LOGO	25.67 (21)	4.87 ± 0.97 (28)	23.66 ± 4.24 (29)	27.78 (4)
L1-LSMI	22.46 (10)	4.33 ± 1.10 (17)	27.54 ± 2.24 (25)	26.39 (5)
IAMB	28.88 (6)	4.80 ± 0.89 (24)	14.73 ± 2.31 (30)	27.78 (3)
HITON	24.06 (22)	4.26 ± 0.66 (28)	28.24 ± 5.26 (23)	29.17 (3)
JMI	24.06 (30)	4.63 ± 0.58 (30)	20.70 ± 3.36 (16)	18.06 (14)
DISR	24.06 (22)	5.00 ± 0.83 (29)	19.86 ± 2.69 (21)	19.44 (4)
QPFS	24.60 (25)	4.37 ± 0.74 (28)	20.21 ± 1.97 (23)	23.61 (12)
CMIM	24.06 (15)	4.61 ± 0.88 (27)	16.06 ± 2.95 (30)	16.67 (24)
CIFE	23.53 (15)	5.89 ± 1.61 (30)	16.83 ± 3.26 (27)	29.17 (11)
MIQ	24.06 (16)	4.83 ± 0.84 (19)	18.52 ± 3.17 (28)	22.22 (15)
SPECCMI	24.06 (27)	4.61 ± 1.14 (28)	20.14 ± 4.67 (29)	19.44 (14)
RRCT	22.99 (18)	4.72 ± 0.56 (29)	13.87 ± 2.48 (27)	13.89 (20)

The design matrices are summarized in the form *N×M* [number of samples × number of features]. When two design matrices are mentioned, the first was used for training (selecting features and training the statistical learner) and the second for testing performance. The presented results are the percentage of misclassification values, and the number in parentheses is the number of features that gave best-performance results searching for results with 1 … min(*M*,30) features (see Figure 2 for details). When the assessment was done using 10-fold cross validation, the results are presented in the form mean ± SD.

**Table 3:** Out of sample RF, the percentage of misclassification for the multi-class classification datasets

	Cardiotocography [2,129 × 21]	Handwriting [2,000 × 649]	Lymph [148 × 18]	SRBCT [63 × 2,308; 25 × 2,308]
GSO	8.87 ± 1.62 (19)	2.00 ± 0.85 (30)	12.16 (14)	25.00 (10)
mRMR Peng	9.10 ± 1.75 (21)	1.05 ± 0.55 (29)	13.51 (15)	0.00 (10)
mRMR Spearman	9.06 ± 2.06 (15)	2.85 ± 1.06 (26)	13.51 (18)	0.00 (25)
Information gain	8.49 ± 2.32 (16)	1.50 ± 1.05 (26)	12.84 (17)	5.00 (18)
RELIEF	9.25 ± 1.54 (21)	5.85 ± 1.43 (29)	13.51 (16)	0.00 (19)
CFS	9.39 ± 2.69 (14)	2.90 ± 1.37 (30)	13.51 (10)	0.00 (9)
CBF	11.75 ± 2.50 (12)	1.45 ± 0.86 (22)	18.92 (4)	0.00 (27)
SIMBA	8.58 ± 1.87 (19)	2.60 ± 1.15 (30)	12.84 (12)	0.00 (6)
LOGO	9.25 ± 2.04 (14)	0.90 ± 0.81 (30)	12.16 (15)	5.00 (12)
L1-LSMI	9.53 ± 2.47 (18)	1.85 ± 1.03 (21)	12.16 (15)	5.00 (7)
IAMB	9.34 ± 2.65 (11)	2.15 ± 0.75 (27)	16.22 (4)	25.00 (10)
HITON	9.48 ± 1.71 (20)	3.90 ± 1.13 (27)	12.84 (18)	15.00 (4)
JMI	8.73 ± 1.38 (16)	1.35 ± 0.58 (30)	13.51 (14)	0.00 (24)
DISR	8.77 ± 1.64 (20)	1.80 ± 1.03 (24)	14.19 (17)	0.00 (23)
QPFS	9.25 ± 2.04 (13)	1.15 ± 0.91 (27)	13.51 (15)	5.00 (18)
CMIM	9.48 ± 2.10 (19)	1.15 ± 0.82 (30)	13.51 (15)	0.00 (27)
CIFE	9.72 ± 1.82 (21)	2.40 ± 0.81 (20)	13.51 (14)	15.00 (11)
MIQ	9.72 ± 1.49 (10)	1.90 ± 0.84 (30)	13.51 (15)	10.00 (30)
SPECCMI	8.87 ± 1.72 (17)	1.70 ± 1.09 (22)	12.84 (17)	0.00 (13)
RRCT	9.53 ± 1.37 (17)	2.30 ± 0.75 (28)	12.84 (15)	0.00 (18)

The design matrices are summarized in the form *N×M* [number of samples × number of features]. When two design matrices are mentioned, the first was used for training (selecting features and training the statistical learner) and the second for testing performance. The presented results are the percentage of misclassification values, and the number in parentheses is the number of features that gave best-performance results searching for results with 1 … min(*M*,30) features (see Figure 3 for details). When the assessment was done using 10-fold cross validation, the results are presented in the form mean ± SD.

Specifically, among the binary classification problems, RRCT is second best for the SPECTF dataset and best for Relathe and ovarian cancer, whereas for Spambase, things are less clear since there is some variability in the reported performance due to the randomness in the 10-fold cross-validation (CV) process (for example, the standard deviation is more than the difference between RRCT and the best-performing FS algorithm). For the ovarian-cancer dataset (which is a fat dataset) in particular, RRCT exhibits consistently better performance compared with all competing FS algorithms. This likely reflects that the interaction component that is computed with RRCT is crucial in this type of biological dataset.

For the multi-class classification datasets, findings were less clear, and again, there was no consistently dominating FS algorithm. Information gain was the best-performing algorithm for cardiotocography, LOGO was best performing for the handwriting dataset, and GSO, LOGO, and L1-LSMI were best performing for the lymph dataset. For the SRBCT dataset, many algorithms classified correctly all samples even though there were some differences in the number of features required to achieve that, and SIMBA should be considered the winner, having achieved that with the lowest number of features, thus reflecting a more parsimonious model. Overall, we remark that RRCT was very competitive for the multi-class classification problems except for the handwriting dataset, where LOGO was clearly better.

### Computational complexity

**Figure 4:**
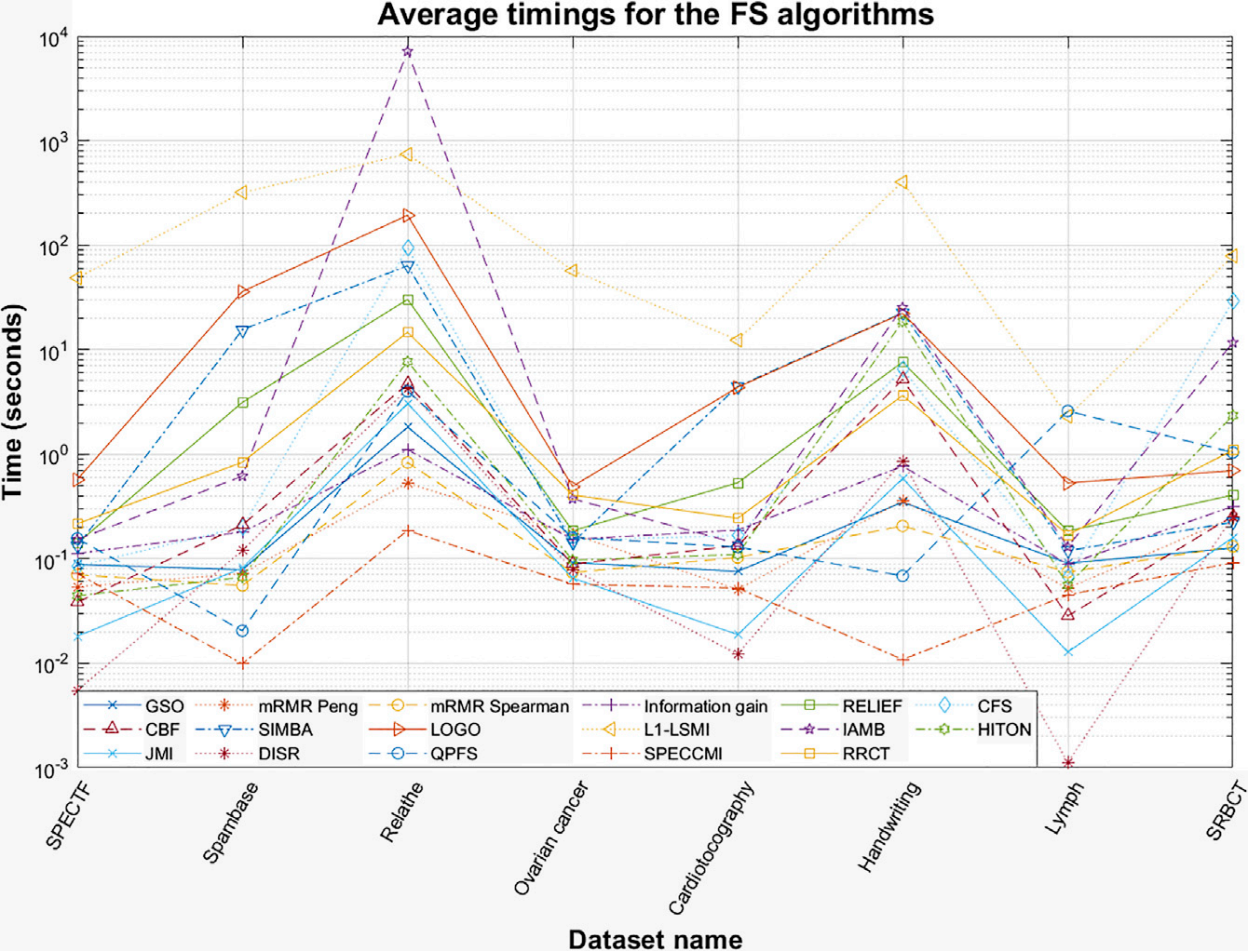
Average timings for the feature-selection (FS) algorithms explored in this study across the 8 real-world datasets The y axis is presented in a logarithmic scale for convenience. All experiments were run on a desktop Windows 10 machine with an Intel i9-9900K CPU at 3.6 GHz with 64 GB RAM using MATLAB 2021b.

This section briefly reviews the computational complexity of the 20 FS algorithms explored in this study. Figure 4 provides the overall average timings to run each of the FS algorithms. Because of the way the functions for QPFS and SPECCMI were coded, the timings for these two algorithms in Figure 4 reflect the required time to run QPFS and MIQ and SPECMI, CMIM, and CIFE, respectively. Overall, almost all algorithms scaled well with a large number of samples and features and provided outputs within a few seconds. L1-LSMI and IAMB were the computationally most-demanding approaches. RRCT is computationally efficient, exhibiting only slightly greater computational burden, e.g., to mRMR Spearman, and, on average, is less computationally intensive compared to the popular FS algorithm RELIEF. For further details, see FS assessment in the experimental procedures, and consult Table 4 for the dataset used in the study.

**Table 4:** Summary of the 12 datasets used in the study

Dataset	Data matrix	Associated task	Type
Synthetic 1 (Breiman)	60 × 30	classification (2 classes)	C (30)
Synthetic 2	1,000 × 100	classification (2 classes)	D (100)
Synthetic 3 (Guyon)	1,000 × 500	classification (10 classes)	C (500)
Synthetic 4 (Guyon)	100 × 500	classification (8 classes)	C (500)
SPECTF^a^	80 × 44; 187 × 44	classification (2 classes)	C (44)
Spambase^a^	4,601 × 57	classification (2 classes)	C (57)
Relathe^b^	1,427 × 4,322	classification (2 classes)	D (4,322)
Ovarian cancer^c^	72 × 592	classification (2 classes)	C (592)
Cardiotocography^a^	2,129 × 21	classification (10 classes)	C (14), D (7)
Handwriting^a^	2,000 × 649	classification (10 classes)	C (649)
Lymph^a^	148 × 18	classification (4 classes)	D (18)
SRBCT^d^	63 × 2,308; 25 × 2,308	classification (4 classes)	C (2,308)

The size of each data matrix is N×M, where N denotes the number of instances (samples) and M denotes the number of features. The last column denotes the type of variables: continuous (C) or discrete (D). Where two design matrices are mentioned, the first is used for training (selecting features and training the statistical learner) and the second data matrix for testing performance.

a Downloaded from the UCI Machine Learning Repository (http://archive.ics.uci.edu/ml/datasets.html).

b Downloaded from the ASU FS Repository (https://jundongl.github.io/scikit-feature/datasets.htmlhttp://archive.ics.uci.edu/ml/datasets.html.)

c Downloaded from http://www.biomedcentral.com/1471-2105/10/259/additional.

d Download from http://www-stat.stanford.edu/∼tibs/ElemStatLearn/.

## Discussion

### Study overview and primary findings

**Figure 5:**
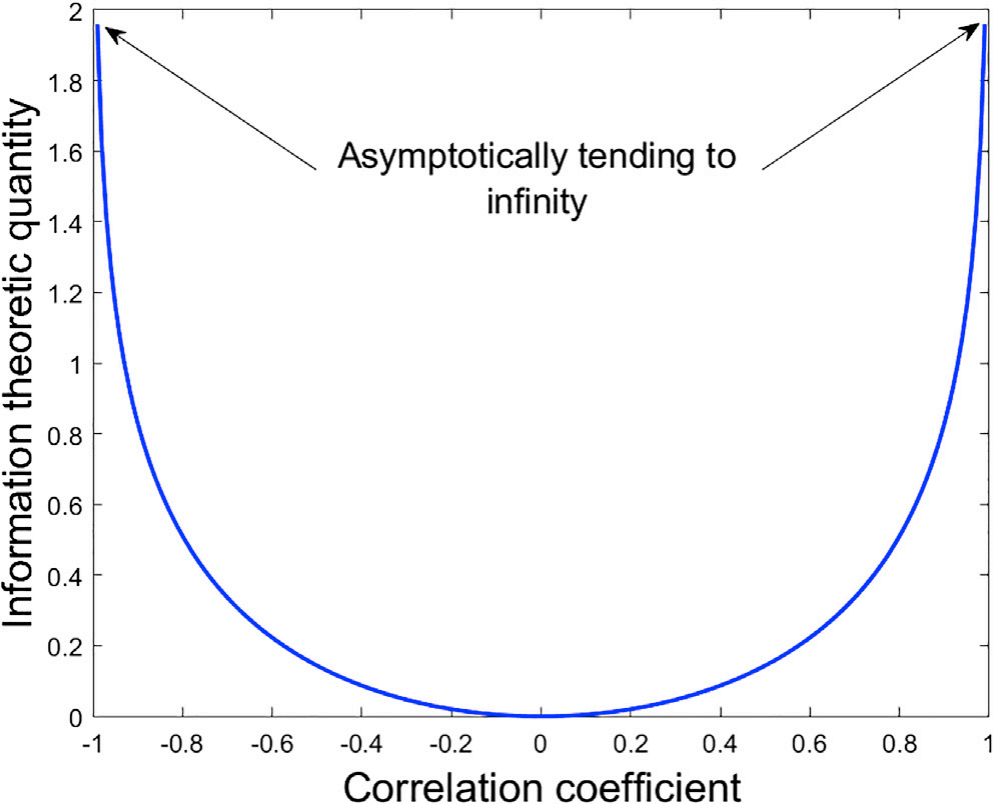
Information theoretic (IT) quantity (relevance or redundancy) as a function of the rank (Spearman) correlation coefficient ρ, computed as $\text{I}\left(\rho \right)=-0.5\cdot \text{log}\left(1-\rho^{2}\right)$ Asymptotically, as the absolute value of the correlation coefficient tends to ±1, the IT quantity becomes infinite (in practice, we set this to a very large value: 1,000).

**Figure 6:**
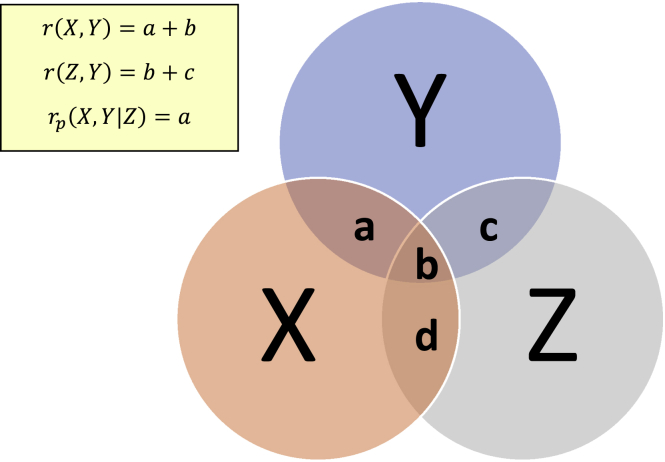
Graphical representation of the effect of the partial correlation coefficient The lowercase letters represent the shared information between the random variables.

We proposed a new filter FS algorithm, RRCT, which was directly inspired by previous theoretical exploration drawing on the three main components required for effective FS: relevance, redundancy, and complementarity (feature interactions). RRCT builds on an intuitively appealing conceptual formulation using partial correlation coefficients and an information-theoretic inspired transformation of rank correlations (see Figures 5 and 6). We investigated the potential of RRCT’s benchmarking performance against 19 filter FS algorithms across four synthetic datasets and eight real-world datasets (see Table 4). The datasets were chosen to be broadly representative of different practical challenges (e.g., very small number of samples, fat datasets, continuous and discrete features, multi-class classification). RRCT was shown to be very robust, in some datasets coming clearly on top and in other datasets performing very well and relatively close to the best-performing algorithms. We demonstrated that by generalizing point estimates of shared information content (quantified via correlation coefficients) and by accounting for multi-variable complementarity (using partial correlation coefficients), we developed a very effective, generic FS algorithm that improves on other classical filter schemes. RRCT is very fast and does not require fine-tuning of parameters, making it a useful tool that can be easily used off the shelf.

### Results in context

A major strength of this study is the empirical comparison of 20 FS algorithms across 12 datasets, whereas most studies in the FS literature typically restrict comparisons to a limited number (often less than 10) or type of FS algorithms, e.g., only MI-based.^13^ We started our empirical exploration by focusing on synthetic datasets, where the ground truth of the contributing features toward estimating the response is known *a priori*. The four synthetically generated datasets investigated here were designed to study well-known practical challenges, including using datasets with a very small number of samples and a very small number of contributing features compared with the overall number (i.e., many probes in a dataset), and an increasingly emerging challenge with a small number of samples for a large number of features in a multi-class classification setting. We found that GSO, IAMB, and RRCT were consistently well suited to discarding probes across the four synthetic datasets, whereas more advanced FS algorithms generally performed poorly, particularly for the fourth synthetic dataset (the fat dataset). Overall, this finding is in agreement with previous suggestions encouraging the careful exploration of FS-algorithmic approaches.^3^^,^^22^, ^23^, ^24^

Similarly, the choice of the eight real-world datasets was driven by identifying types of challenges and using datasets, many of which had been previously used in the FS literature. Findings were less decisively clear compared with the synthetic datasets in terms of FS algorithm performance inferred by the RF outputs. We remark that there is no clearly superior FS algorithm for all datasets, which can be seen as one manifestation of the no free lunch theorem: no one method dominates all others over all possible datasets. This is something that previous studies in FS have similarly reported, indicatively.^3^^,^^9^^,^^10^^,^^13^

Exploring the FS algorithms in more detail side by side, GSO came out on top for the lymph dataset; however, its performance was much worse in datasets with a smaller number of samples compared with the best-performing FS algorithms (particularly for the two fat datasets). We believe that GSO is an FS algorithm that should be more carefully considered in practical applications when having sufficiently large sample sizes, e.g., this verifies a further large-scale application where GSO performed well compared with other FS algorithms.^25^ The mRMR Peng algorithm is probably one of the most popular FS algorithms in practice. Overall, we found that mRMR Peng was reasonably competitive, although, again, its performance degraded in two of the fat datasets (fourth synthetic dataset and ovarian-cancer dataset). On the other hand, it was among the FS algorithms that led to zero misclassifications on SRBCT (the second real-world fat dataset). It performed well on datasets with a large number of samples (Spambase, Relathe, cardiotocography, handwriting), which is unsurprising given that it relies on the computation of the MI. From a theoretical perspective, mRMR (as a concept) does not take complementarity into account, so it is likely that this is what caused its relatively lower performance, e.g., in the SPECTF and ovarian-cancer datasets. mRMR Spearman was reasonably competitive and even better performing that mRMR Peng for the datasets with small sample sizes, including for the fat datasets. Unsurprisingly, in datasets with a larger number of samples, mRMR Peng had a clear edge (especially the Relathe and handwriting datasets). Therefore, as expected, mRMR Spearman can be considered a computationally simpler approach that should probably only be preferred over mRMR Peng on small-sample-size studies; intuitively, this makes sense, because the computation of the correlation coefficient is more robust compared with the computation of MI. We empirically verified that MIQ, which is based on the same principle and basic formation as mRMR (the only difference being that the criterion operates on the ratio of relevance over average redundancy rather than their difference as in mRMR), has been justifiably overshadowed by mRMR since it performed worse by comparison. L1-LSMI has performed well overall, coming out on top for the SPECTF and lymph datasets. However, for the Relathe and ovarian-cancer datasets, it was still considerably worse than many of its competitors. We can tentatively infer that L1-LSMI is an algorithm that should be considered primarily in settings with a sufficiently large sample size (which is intuitively expected as it is an MI-based approach) and not in very sparse datasets where, again, the computation of the squared MI would be challenging. RELIEF is another popular FS algorithm; however, its performance overall was worse compared with some of the more successful FS algorithms, particularly where redundancy is a key property that needs to be accounted for (e.g., the handwriting dataset). Instead, the conceptually similar nearest hit (NH)- and nearest miss (NM)-based approaches, SIMBA and LOGO (the latter especially), performed better. LOGO was the top algorithm for handwriting and lymph and generally performed well in the real-world datasets, apart from the ovarian-cancer dataset. Therefore, LOGO appears to operate well in large datasets with many noisy or redundant continuous features. It is possible LOGO could be adjusted to perform better by changing the distance metric internally used as a hyper-parameter (e.g., for the Relathe dataset); however, we wanted to keep to the premise of working on vanilla-based implementations. The FS approaches aiming to determine Markov blankets (IAMB and HITON) had very variable performance: HITON was the top-performing algorithm for Spambase (largest dataset in terms of number of samples investigated here). However, it did not perform well for the other large datasets (Relathe and handwriting) which also comprise a very large number of features. Therefore, we tentatively suggest that HITON might be a good approach to determine the Markov blanket of the response in datasets with a relatively limited number of features. On the contrary, IAMB appeared to perform better in those settings with a large number of features compared with HITON.

The algorithmic approaches aiming to address FS globally (QPFS and SPECCMI), although theoretically well-founded, in practice did not demonstrate any superiority in terms of the simpler-based filter FS approaches in the different types of problems investigated here. By design, these FS algorithms aim to compute high-dimensional interactions among the features and the response, and hence, we can anticipate that they will likely require a large number of samples to operate well. Some of the practical challenges with QPFS have been previously discussed in Vinh et al.,^26^ including not adapting well to datasets with a small number of samples because the MI cannot be efficiently computed. This is something that we verified in this study, e.g., with the first synthetic dataset where both methods selected many false features, whereas they both were more competitive against competing FS algorithms when presented with thousands of samples (e.g., Spambase and handwriting). Moreover, we have found that these global approaches are also likely not performing well in sparse datasets (see results for Relathe), which again can be attributed to practical challenges in accurately computing higher-order interactions aiming to jointly find a predictive feature subset. From a practical perspective, an additional challenge with QPFS and SPECCMI is that we need to discretize the continuous features in order to compute high-dimensional feature interactions computationally efficiently, which adds another level of complexity.

We remark that many of the advanced FS schemes examined here (including QPFS and SPECCMI) rely on MI, and its computation is very challenging in practice, particularly in relatively small- to middle-sized sample datasets for continuous features.^13^^,^^27^^,^^28^ This is why, in practice, most FS algorithms first discretize the continuous features and operate on the discretized features.^13^^,^^26^ There is no single rule of thumb on the number of discretized feature values or indeed the approach that should be used to discretize features (e.g., univariate or multivariate), and in practice, this was usually predefined to be a single number, e.g., in the context of FS, this was usually taken to be 5.^13^^,^^26^ This topic is of broader interest in ML^29^ and is an area that needs to be explored further in combination with MI-based FS algorithms.

Previously, Brown et al.,^13^ in their extensive MI-based FS investigation, reported that JMI is their recommended algorithm, at least for relatively small data samples, which was generally in agreement with our findings here for MI-based approaches. We found that JMI can indeed be competitive in some datasets (e.g., we note its good performance in the handwriting and lymph datasets); however, overall, there were non-MI-based filters that were better performing.

RRCT was the clear winner in the Relathe and ovarian-cancer datasets and was tied with a few competing algorithms on the SRBCT dataset, while generally performing well across datasets. RRCT is well suited to datasets where feature complementarity is prominent, such as in micro-array datasets (ovarian cancer and SRBCT). It also outperformed competing algorithms in the sparse dataset (Relathe) because, intrinsically, this is well handled using correlation coefficients, and joint interactions with sparse data can be captured similarly well using the partial correlation coefficients. Since it is a correlation-based filter, its weakness is in datasets where the relationship between features and the response can only be captured by higher-order moments (e.g., the cardiotocography and handwriting datasets). Therefore, when there is a dataset with a fairly large sample size available and continuous features with likely non-linear underlying statistical relationships (with the response and within features), it is likely that an FS method such as LOGO would provide better results. We emphasize that RRCT has the desirable practical property that it is robust: it will generally be reasonably competitive even when not performing among the best FS algorithms for a particular dataset, whereas some of the competing FS algorithms are considerably more variable in their resulting performance across datasets. Indicatively, see HITON, which may be very good in some datasets (top algorithm in Spambase) and very bad performance in others (e.g., Relathe and ovarian cancer). Moreover, although not tested in this study, RRCT has the additional advantage that it can be readily deployed for both classification and regression applications, whereas some of the investigated FS algorithms cannot be readily generalized to such settings.

To account for inherent variability in FS when used in a CV setting and before presenting the selected feature subsets into the statistical learner, we used a robust voting strategy (see FS strategy and Table 5). As we argued previously,^15^^,^^30^^,^^31^ this voting strategy approach ensures we can decide on the feature subset that can be used on the basis of perturbed versions of the original dataset and is applicable out-of-the-box with any greedy FS algorithm. We stress this is an important step to enable side-by-side comparisons of the selected feature subsets across FS algorithms in terms of FS consistency, which could be further explored.

**Table 5:** Proposed methodology for selecting features using the greedy feature-selection algorithms and a voting strategy

**Input:**$X\;\varepsilon \;R^{N\times M}$ and $y\;\varepsilon \;R^{N\times 1}$, where N is the number of data samples and M is the number of features. Depending on the FS algorithm, you may need additional hyper-parameters and/or pre-processing of the data (e.g., discretization). **Process** 1. For each FS algorithm, create an empty set S, which will contain the indices of the selected features. 2. Randomly select 90% of the data samples from the data matrix X along with their responses, y. 3. Run the FS algorithm using the 90% randomly selected samples. The result is an ordered sequence of features (often you can choose the number of features m≤M as the output to save on computational time), where the first feature is considered the most important for the chosen FS algorithm. 4. Repeat steps 2 and 3 multiple times, say $R_{p}$, and store the results in a matrix $X_{FS}$ (of size $R_{p}\times \;m$), In each of the $1\ldots R_{p}$ rows of $X_{FS}$, we store the selected feature subset. 5. Voting to decide on the final feature subset for each FS algorithm: feature indices are incrementally included, one at a time, in S. For each of the 1…m steps, we find the indices corresponding to the features selected until that step for all the repetitions in step 4 (i.e., use the $R_{p}\times \;L$ subset of $X_{FS}$, where L corresponds to the features selected in the first L FS steps [in the last step L=m]). 6. We select the feature index that appears most frequently among these $R_{p}\times L$ elements and that is also not already included in S. This index is now included as the L^th^ element in S. Ties are resolved by including the lowest index number. 7. Repeat steps 5 and 6 for the number of features we want to ultimately use (i.e., m). **Output:**$\varphi_{s}\;\varepsilon \;R^{1\times m}$ vector with the ordered sequence of selected features in descending order of importance. The indices in this sequence correspond to the columns in the data matrix X.

In terms of computational demands (see Figure 4), we found that most FS algorithms generally provided feature ranking within a few seconds for almost all investigated datasets here. L1-LSMI was, in general, the computationally most-demanding algorithm, with IAMB surpassing it for the Relathe dataset, i.e. IAMB scales relatively poorly with a large number of features. RRCT scales very well, and for the Relathe dataset, it provided outputs within 14 s; indicatively, it was almost always faster compared with RELIEF running within a few seconds, so it can be considered a computationally very efficient FS approach.

### Limitations and future work

The main limitation of RRCT is that it cannot quantify highly non-linear statistical relationships between variables. This is by design as a practical trade off to develop a computationally efficient, robust, parameter-free FS algorithm. RRCT implicitly considers that statistical relationships between pairs of variables can be adequately captured using monotonic relationships for the relevance and redundancy terms (quantified using the Spearman correlation coefficient), which could be considered a major simplification for practical real-world problems. Along these lines, the information theoretic (IT) formulation of RRCT presumes that the underlying distributions are quasi-Gaussian, which seems rather restrictive and rigid. Nevertheless, across machine-learning applications, many theoretically strong assumptions actually still lead to surprisingly good results in practice. One example is with principal-component analysis (PCA),^1^ which often leads to generally good outcomes in feature transformation applications even though it makes quite restrictive assumptions in terms of the underlying data relationships compared with alternatives.^32^ Conceptually, PCA only operates on the first two central-order moments (mean and variance), thus intrinsically not considering more complicated aspects of the statistical distributions for the variable relationships (linearity assumption). Similarly, the Naive Bayes classifier works surprisingly well given the rigid assumption of feature independence (which typically does not hold in practical applications).^33^^,^^34^ Intuitively, prior transformation of the continuous features to shape into more quasi-normal distributions suggests itself as a potentially useful pre-processing step before feeding features into RRCT. Power transformations of the features, of which the Box-Cox transform^35^ is one of the most popular, is therefore an appealing option to explore. In our experiments (data not shown), applying the Box-Cox transformation across continuous features, which were not normally distributed, did not seem to lead to consistent performance improvement across datasets, and we did not find a theoretical or empirical rule to decide on when this could be effectively employed. It is likely that this is not straightforward because the maximum likelihood approach used for the power transformation is challenging in the presence of outliers; this is an area that could be explored further, e.g., along the lines of Marazzi and Yohai.^36^ Other density-normalization techniques might be more appropriate pre-processing steps prior to FS with RRCT (and possibly other FS algorithms), which could be explored in future work.

We attribute the overall success of RRCT across many of the practical problems despite its simplifying assumptions regarding the underlying variable distributions to the combination of three key components: (1) using the ranks within variables (implicitly used when computing rank correlation coefficients such as Spearman, thus overcoming certain problems with variable distributions, e.g., outliers skewing correlations), (2) the non-linear IT transformation of the correlation coefficients to form the relevance and redundancy terms, and (3) the complementarity term, which quantifies higher-order feature interactions toward estimating the response. Future work could explore building on the RRCT framework by integrating a different statistical-association measure instead of the Spearman correlation coefficient so that it can capture more general statistical relationships (although this would introduce additional computational complexity).

We attempted to provide plausible explanations as to why particular FS algorithms might be suited to specific datasets (or domains), which might give a good indication of their performance in similar settings, e.g., in terms of the number of classes, features, and samples (or possibly their ratio). This study has provided some useful tentative insights comparing a large range of filter FS algorithms across different indicative types of problems; however, we stress that these findings need to be evaluated more extensively. A very large empirical study using diverse datasets to identify the settings where particular FS algorithms excel and fail would be very useful in this regard.

It is useful to consider FS within the wider context of data processing and see how it fits with other methods that may be complementary in related tasks. Some FS algorithms, including some examined in this study, aim to provide feature weights toward determining the relative importance of features for a statistical learning task (e.g., RELIEF, SIMBA, LOGO). Although usually all samples are assigned equal weight, under certain circumstances, we may want to vary those weights to assign relative importance to different samples. Applying similar reasoning to FS, some research work has explored ways toward assigning different weights to samples rather than features. Inverse probability weighting (IPW) is one approach that can be used to achieve that, and we refer to Mansournia and Altman^37^ for indicative applications. In principle, it would be possible to jointly explore IPW and FS, e.g., in unbalanced high-dimensional problems to assign weights both to samples and features toward specific statistical-learning tasks. Another research area that has attracted considerable research interest is interpretable machine learning. FS is a step toward facilitating interpretability by reducing the number of features presented into a statistical learner and hence enabling researchers to focus on interpreting the key characteristics and properties of the modeled system or the process characterized by the chosen lower-dimensional feature set. In addition to this, the research community has developed methods toward interpreting the outputs of statistical-learning models, and a convenient unified framework is provided using Shapley Additive Explanations (SHAP).^38^ SHAP assigns each feature an importance score for a particular estimate for the statistical-learning model used (e.g., a trained RF), i.e., SHAP values explain how features impact the output of the model. Therefore, we can view FS as the first step in identifying the feature subset that is presented into a statistical learner and SHAP as the post-processing method to interpret the local feature importance toward the model output for a specific query sample. Future work could investigate further the synergies between FS and SHAP toward providing a streamlined approach to building parsimonious, robust, and interpretable models.

### Concluding remarks

Collectively, this study’s findings indicate that RRCT is a very competitive algorithm across diverse settings, can be readily deployed when having datasets with mixed-type variables without any further pre-processing, and is directly applicable in both classification and regression applications (although in this study, we have only focused on classification applications because many of the FS algorithms explored herein only operate in classification). We envisage RRCT finding use as a robust, computationally efficient, off-the-shelf FS algorithm across any type of dataset, particularly in datasets with a small-medium number of samples and in fat datasets. Similarly, we provided some insights and tentative ideas regarding the investigated FS algorithms and the settings where they may (or not) be well suited for a range of typical types of data problems.

## Experimental procedures

### Resource availability

#### Lead contact

The lead contact is Athanasios Tsanas (Thanasis; atsanas@ed.ac.uk and tsanasthanasis@gmail.com).

#### Materials availability

This work generated no non-code materials.

### Hardware and software

All experiments were run on a desktop Windows 10 machine with an Intel i9-9900K CPU at 3.6 GHz with 64 GB RAM. The source code has been tested on MATLAB 2021b.

### Datasets

Table 4 summarizes the datasets used in the study for easy reference. The key information herein is presented in terms of the (1) size of the data matrix in the form number of samples *N* × number of features *M*, (2) associated task (regression or classification), and (3) variable type (discrete or continuous variables, indicating where there is a data matrix with mixed-type variables). Overall, we used 4 synthetic datasets and 8 real-world datasets to evaluate the performance of the FS algorithms.

#### Synthetic data

The rationale for the choice of the synthetic datasets was to explore the performance of the 20 FS algorithms across indicative types of problems that may be considered broadly representative of what can be seen in practice (datasets with a small number of samples, datasets with binary features, fat datasets, multi-class classification datasets). Evaluating the performance of the FS algorithms in these synthetic datasets where we know *a priori* the true features provides direct insights on how well they can recover the minimal feature set that is jointly predictive of the response and can serve to highlight types of problems where specific FS algorithms would likely not perform well. For transparency, we used established synthetic data generators in three out of the four synthetic datasets.

The first synthetic dataset was generated using Breiman’s sample generator, which produces 60 samples and 30 features drawn from a multivariate normal distribution, where the resulting continuous response was binarized using a quantile-based transformation to obtain a balanced binary classification dataset. Only 3 features contribute to the response (true features), and the remaining 27 features are probes (false features). The challenge with this dataset is that there are very few samples, so methods that rely on large sample sizes for their internal selection criteria would likely not perform well. Therefore, the underlying motivation for starting our empirical exploration with this dataset was to evaluate how well the 20 FS algorithms operate on an indicative dataset with limited samples.

The second synthetic dataset was generated to assess how well FS algorithms cope with binary features. Specifically, we generated a dataset with 1,000 samples and 100 uniformly sampled binary features. The response was computed as $y=\frac{1}{5}\sum_{i=11}^{15}X_{i}-X_{16}+X_{17}\cdot X_{18}$, which was subsequently binarized using quantile-based transformation to obtain a balanced binary dataset. The underlying motivation to create the second synthetic dataset was to explore whether the setting with exclusively binary features (this type of dataset occurs in some fields of medicine and other practical problems) poses challenges for FS algorithms in recovering the true features.

The third and fourth synthetic datasets were generated using Guyon’s sample generator, which we have adapted to extend its use toward generating multi-class classification outputs. The generator requires users to specify the numbers of samples, of independent features (a subset of which is the useful features, i.e., they are used for the statistical-learning task), and of linearly dependent features upon the independent features. Guyon’s generator draws independent features normally distributed with additive white Gaussian noise (standard deviation = 0.1), and the features are shifted and re-scaled randomly to span three orders of magnitude. The response was computed by multiplying the useful features with a random weight vector, the components of which were drawn from a normal distribution, and quantizing the result using quantile-based transformation to obtain balanced datasets. The third synthetic dataset comprises 1,000 samples and 500 features, of which 10 are useful (predictive of the response) for a 10-class classification problem. The motivation for including the third dataset was to assess whether FS algorithms can perform well in a high-dimensional multi-class classification problem toward recovering the true features. Finally, the fourth synthetic dataset comprises only 100 samples and 500 features (of which 10 are useful) for an 8-class classification problem to test the performance of FS algorithms with an indicative fat dataset. Fat datasets are met increasingly frequently in different practical applications (e.g., with micro-array datasets), and therefore the motivation for generating the fourth synthetic dataset was to assess whether the FS algorithms can correctly recover the true features in such a challenging problem.

For convenience when working on the synthetic datasets, we use the terms true features to refer to the feature set that is jointly the minimal subset of features predicting the response and false features to refer to the remaining features.

#### Real-world data

Similar to the synthetic datasets, the rationale for the choice of the real-world datasets used in this study was to explore the performance of the 20 FS algorithms across indicative types of problems that may be considered broadly representative. By pursuing this empirical investigation, we gain insight into the types of problems where specific FS algorithms perform well (or not) and hence can tentatively draw conclusions on which FS algorithm(s) we may opt to use in related types of problems. This empirical exploration serves to help better understand challenges and limitations FS algorithms may have when presented with complicated real-world problems, beyond the carefully generated synthetic datasets introduced in the preceding section. Specifically, we wanted to explore indicative sparse datasets, fat datasets, and datasets with a varying number of samples and features. We used 8 indicative datasets that reflect a range of practical settings, including binary classification and multi-class classification problems, and datasets that range from a few samples and features to thousands of samples and features. Many of these datasets have been previously used in FS research literature.

The SPECTF dataset focuses on heart disease by processing cardiac single-proton emission computed tomography (SPECT) images, where each sample has been clinically assessed as normal or abnormal. The features have been extracted by processing the SPECT images by focusing on specific clinical regions of interest. This is a dataset that has been often used in FS research literature, and the rationale for including it in this empirical investigation is that we have a relatively limited number of samples in the training set that we use to select features, a setting that may be challenging for FS algorithms (akin to the first synthetic dataset).

The Spambase dataset contains 57 features to characterize a collection of emails coming from filed work and personal emails. The binary response denotes whether the e-mail was considered spam (1) or not (0), i.e., an unsolicited commercial email. The underlying motivation for including this dataset is that it represents the type of problem where we have a large number of samples and a relatively small number of features.

The Relathe dataset is a large sparse dataset with discrete and binary features characterizing text. The rationale for including this challenging dataset is both because of the very large number of features and because it represents a sparse problem (which is representative of some specific applications).

The ovarian-cancer dataset is one of the two real-world fat datasets used in this study. The features that have been extracted characterize metabolomic data produced from sera of ovarian-cancer patients and benign control participants. For details, we refer to Guan et al.^39^ Similar to the fourth synthetic dataset, the rationale for including this dataset in the analysis is that it represents an emerging type of problem, which is increasingly seen with relatively few samples and a large number of features.

The cardiotocography dataset contains information from processing fetal cardiotocograms. They were assessed by three expert obstetricians, and a consensus classification label of the pattern class code constitutes the 10-class response. The motivation for including this dataset was to use it as a representative type of problem with a large number of samples in an unbalanced multi-class classification setting.

The handwriting dataset consists of 649 features extracted from a collection of Dutch utility maps to identify handwritten numerals (0–9). This is a nicely balanced dataset with 200 samples per class. The motivation for including this dataset is because we can use it to compare findings against the cardiotocography dataset given that we have a similar number of samples and classes, whereas handwriting contains a large number of features.

The lymphography dataset (abbreviated as “lymph” here to conform with other studies in the machine-learning literature) is derived from the field of oncology and comprises binary and multi-level discrete features aiming to estimate a four-class response: normal find, metastases, malign lymph, and fibrosis. The motivation for including this dataset was to explore how well FS algorithms might perform in a type of problem with relatively few samples and discrete features in a multi-class classification setting.

The SRBCT dataset is derived from a set of micro-array experiments where samples arose from small, round blue-cell tumors (SRBCTs) found in children, which have been classified into four major types: Burkitt lymphoma (BL), Ewing’s sarcoma (EWS), neuroblastoma (NB), and rhabdomyosarcoma (RMS). The dataset has been used as an indicative fat dataset in the standard statistical-learning book by Hastie et al.^1^ The motivation for including SRBCT was similar to the use of the fourth synthetic dataset and the ovarian-cancer dataset: to explore the performance of FS algorithms in another fat dataset (compared with ovarian cancer, which represented a binary-class classification problem, this represents a multi-class classification problem).

Overall, these datasets provide good indicative examples of diverse types of data problems to assess the performance of FS algorithms.

### FS

This section provides some background on the terminology and key concepts in FS and summarizes the FS algorithms used in the study, including the development of the new FS algorithm proposed herein (RRCT), along with the FS strategy.

#### Key terminology in FS and main concepts of filter approaches

Given the data matrix (or design matrix) $X\;\varepsilon \;R^{N\times M}$ and the response $y\;\varepsilon \;R^{N\times 1}$, where N is the number of samples (instances) and M is the number of features, the FS algorithms aim to reduce the input feature space M into m features, where m<M (m can be chosen based on prior knowledge and possible constraints of the application or can be determined via CV). That is, we want to select a feature set S comprising m features $\;\left\{f_{j}\right\}\;j\;\varepsilon \;\left(1\ldots M\right)$, where each $f_{j}$ is a column vector in the data matrix X. The optimal feature subset maximizes the joint information content of the selected features with respect to the response. However, this is a complex combinatorial problem, and the optimal solution can only be found by a brute-force search. Since a brute-force search is extremely computationally demanding, particularly for large datasets, sub-optimal alternatives are typically sought. Although in principle combinatorial optimization methods (e.g., genetic algorithms) can be applied to the FS problem, these techniques are also computationally expensive.

As an approximate solution to the combinatorial one, researchers often assess each feature individually in order to determine the overall information content of the feature subset from each individual feature in the subset. There are two FS approaches to incrementally decide on the selected feature subset, one step at a time: (1) sequential-forward process (features are sequentially added to the selected feature subset) and (2) backward elimination (starting from the entire feature set and eliminating one feature at each step). Forward FS is often used in many filter applications because it is computationally more efficient than backward elimination^3^^,^^7^^,^^23^^,^^40^ and is particularly suitable for those problems where we want to reduce a dataset comprising many features to a dataset with a fairly small number of features.

One of the simplest FS algorithms is to use only those features that are maximally related to the response, where the association strength of the features with the response can be quantified using a suitable criterion, I(⋅) (not necessarily a distance metric in the mathematical sense). One straightforward criterion is the Pearson correlation coefficient: this assumes that the association strength between the response and each of the features can be characterized using the mean and covariance (first two joint statistical moments) alone and that the higher-order moments are zero or at least sufficiently small enough that they can be neglected. Alternatively, the Spearman rank correlation coefficient, which is a more general criterion, can be used to quantify the relationship between each feature and the response. More advanced criteria can also be used to characterize potentially non-linear (and non-monotonic) relationships between the features and the response, such as MI. In fact, MI has attracted extensive and systematic interest in the FS literature.^40^, ^41^, ^42^ However, the computation of MI is computationally intensive, particularly in domains with continuous variables.^5^ Conceptually, the simple approach discussed thus far, which relies solely on the association strength between individual features and the response variable, works well in the presence of independent (orthogonal) features. It is now well established that in most practical applications, a good feature subset needs to account for overlapping information shared among features useful in predicting the response.^3^

There are three main concepts that researchers working in this field typically consider: relevance, redundancy, and feature interaction (also known as complementarity or conditional relevance). The first term, relevance*,* is defined as the univariate association strength of a feature with the response, which can be expressed using any approach that can express the statistical relationship between two variables (e.g., correlation coefficients, MI, etc.) and ideally needs to be maximized. The second term, redundancy, refers to the overlapping information shared among features in the feature subset toward predicting the response^3^^,^^4^^,^^23^ and ideally needs to be minimized. The third key concept, interaction, or complementarity, quantifies the extent to which two or more features are strongly associated with the response variable jointly (it is possible the same features may be only moderately associated with the response individually). This third concept was previously largely ignored by many FS algorithms; however, it has been explicitly considered in a number of recent studies, both from a theoretical perspective^13^ and also in practical FS implementations.^26^^,^^43^^,^^44^ An intuitive way to motivate the use of feature interaction is that, in practical problems, it may be that the combination of two or more features is (highly) predictive of the response, whereas single features on their own may or may not be (highly) predictive. An extreme example that is well known comes from Boolean algebra with the use of the “exclusive OR” function (commonly referred to as “XOR”). Readers not familiar with this area can look into the truth table of the XOR function with two or more inputs in any standard textbook or website on Boolean algebra (for example, see the book by Whitesitt^45^). In the XOR example, each feature (in Boolean algebra terminology, features are typically referred to as inputs) is not predictive of the response (in Boolean algebra, the response is referred to as output). On the contrary, if we jointly consider together the (two or more) inputs in the XOR function, we can perfectly estimate the response. Similarly, it can be intuitively understood that in some applications, the joint consideration of two or more features may be more predictive of the response than their individual parts (for example, in biology one gene mutation might not be leading to a harmful phenotype; however, a gene mutation or deficiency that appears jointly across genes may be leading to an adverse outcome). From a computational perspective, it is more challenging to compute the feature-interaction component compared with relevance and redundancy because it requires an algorithmic expression that considers multiple variables at the same time. For example, if using an algorithmic approach that operates on densities, it is computationally much easier to compute marginal densities and conditional densities with two variables (e.g., a feature and the response) rather than joint densities and high-dimensional conditional densities for the features explored. We will see in the following section how FS algorithms, including the new FS algorithm proposed in this study, attempt to overcome these challenges by different computational means.

For further background on FS concepts, we refer to some standard articles in the topic.^3^^,^^10^^,^^46^

#### Known FS algorithms for performance benchmarking

This section briefly summarizes 19 filter FS algorithms, many of which have been widely used in practical applications to determine feature subsets. The aim here was to evaluate how well these FS algorithms perform across indicative synthetic and real-world data problems, against which we can benchmark performance of RRCT, the new FS algorithm that will be introduced in the following section. Due to space constraints, we keep this section brief, summarizing the FS algorithms and their key properties and refer interested readers to the cited research literature for further details. Many of the MI-based FS algorithms have been brought under a unifying framework in Brown et al.;^13^ we also refer to a technical report that summarizes some of the FS methods used herein as part of the ASU FS repository.^47^

The Gram-Schmidt orthogonalization (GSO) algorithm incrementally selects features at each step on the basis of being maximally correlated with the response and minimally correlated with the existing feature subset. The GSO algorithm projects the candidate features for selection at each step onto the null space of those features already selected in previous steps: the feature that is maximally correlated with the target in that projection is selected next. The procedure iterates until the number of desired features has been selected. Further details of the GSO algorithm used for FS can be found in Stoppiglia et al.^48^ and Guyon et al.^3^

Information gain is another generic concept (like GSO) that has been adopted toward FS.^47^ It aims to identify the feature that maximizes the information gained by including it in the feature set compared with the joint information by the already selected features.

The correlation-based filter (CBF)^49^ extends the concept of information gain using the symmetrical uncertainty (normalizing information gain by the sum of entropies) to mitigate bias favoring multi-level features. The CFS is another heuristic FS approach that also relies on symmetrical uncertainty to evaluate the amount of additional information adding a candidate feature brings to the already selected feature subset.

RELIEF was proposed by Kira and Rendell^8^ as a heuristic FS algorithm for binary classification applications, extended to multi-class classification applications by Kononenko,^50^ and later investigated more thoroughly by Robnik-Sikonja and Kononenko.^51^ RELIEF is a feature-weighting algorithm, where each feature is assigned a weight depending on how “useful” it is in the context of predicting the response. Conceptually, features that do not contribute toward predicting the response will be associated with very small weights. The principle of RELIEF is similar to the k-nearest neighbor classifier, making use of the concept of NH and NM. RELIEF is related to hypothesis margin maximization, a concept that is also central in other machine-learning algorithms such as support-vector machines.^52^ RELIEF intrinsically takes feature interactions into account toward their contribution to the separation of samples into differing classes; however, it does not explicitly integrate a mechanism to address redundancy.

$$\begin{array}{l} w\left(f_{j}\right)\overset{\text{def}}{=}\frac{1}{q}\;\sum_{i=1}^{q}\{\underset{Nearest\;hit\;term\;distance}{\underset{︸}{-\frac{1}{\left|\text{NH}\left(x_{i}\right)\right|}\cdot \sum_{x_{n}\;\in \;\text{NH}\left(x_{i}\right)}\left\|x_{i,j}-x_{n,j}\right\|}}\\ +\underset{Normalizing\;factor\;with\;prior\;probabilities}{\underset{︸}{\sum_{y_{l}\neq y_{i}}\frac{1}{\left|\text{NM}\left(x_{i}\right)\right|}\cdot \frac{\text{P}\left(y=y_{l}\right)}{1-\text{P}\left(y=y_{i}\right)}}}\cdot \underset{Nearest\;miss\;term\;distance}{\underset{︸}{\sum_{x_{n}\;\in \;\text{NM}\left(x_{i}\right)}\left\|x_{i,j}-x_{n,j}\right\|}}\} \end{array},$$

where $w\left(f_{j}\right)$ refers to the weight associated with the *j*^th^ feature, q represents the number of instances randomly sampled from the data, $x_{i}$ refers to a data sample (row in the data matrix X), |⋅| refers to the size of NHs or NMs, ‖⋅‖ is a distance metric (the Euclidean or Manhattan distance are typically used). The size of NHs $\left|\text{NH}\left(x_{i}\right)\right|$ and the size of NMs $\left|\text{NM}\left(x_{i}\right)\right|$ are fixed to some pre-specified value, e.g., 10.

SIMBA is an extension of RELIEF and was developed by Gilad-Bachrach et al.^52^ RELIEF does not iteratively re-evaluate the distances in the computation of the weight vector, i.e., the nearest neighbors of a sample are pre-defined in the original feature space. SIMBA integrates the iteratively computed feature weights in its computation of NHs and NMs, thus being more adaptive in re-evaluating sample distances and thus accounting for local information. However, this means that the convex-optimization problem that is being solved with RELIEF becomes a constrained non-linear optimization problem in SIMBA, which poses practical challenges and risks in identifying local minima.

LOGO^7^ aims to decompose the intractable, exhaustive combinatorial problem of FS into a set of locally linear problems through local learning and can be thought of as an extension of RELIEF and SIMBA, with the key concept being to identify NHs and NMs. The local linearization of the global problem of selecting the most appropriate features for predicting the response stems from the use of a margin function, which focuses on the neighborhood of the investigated data samples. The probabilities of hit or miss are obtained from probability density functions, which are computed using kernel density estimation.^1^ LOGO also uses a regularization parameter with the L1-norm to induce sparsity in the resulting weights.

The iterative associative Markov blanket (IAMB) algorithm^53^ is a heuristic approach aiming to identify the Markov blanket (MB) of the response (defined as the set of features conditioned on which all other features are probabilistically independent of the response). IAMB is a sequential-search algorithm that considers features one by one for addition or removal, which more recently was shown to be a greedy iterative maximization of the conditional likelihood.^13^ The underlying assumption in the IAMB algorithmic family is that the data is faithful to some unknown Bayesian network. HITON is a related FS algorithm developed by the same research team that also aims to identify the MB without requiring a large sample size.^54^

The minimum redundancy maximum relevance (mRMR) explicitly takes into account relevance and redundancy using an empirical form where the relevance is computed using the MI between each candidate feature and the response and the relevance is computed using the mean of the pairwise MI between features.^40^ Specifically, it takes the form:

$$\text{mRMR}\overset{\text{def}}{=}\underset{j\;\in \;Q-S}{\text{max}}\left[\underset{relevance}{\underset{︸}{I\left(f_{j},y\right)}}-\underset{redundancy}{\underset{︸}{\frac{1}{\left|S\right|}\sum_{s\;\in \;S}I\left(f_{j},f_{s}\right)}}\right],$$

where $f_{j}$ denotes the *j*^th^ variable in the initial M-dimensional feature space, $f_{s}$ is a variable that has been already selected in the feature index subset S, s is an integer, Q contains the indices of all the features in the initial feature space, that is, 1 … M, S contains the indices of selected features, Q−S denotes the indices of the features not in the selected subset, and |S| denotes the cardinality of the selected subset. I(⋅) is the criterion used, which, for the original mRMR, is the MI. In this study, we used the original mRMR developer’s implementation for the mRMR, which discretizes features using adaptive histograms, and refer to this FS algorithm as *mRMR Peng*. Using the mRMR formula, we can apply a different criterion for determining features, and a computationally very-efficient approach is to use the Spearman correlation coefficient instead.^55^ Hence, we refer to that algorithm as *mRMR Spearman*. A modification of mRMR is using the quotient (ratio) instead of the difference between relevance and redundancy, which gives rise to the MIQ FS algorithm.^56^

An alternative approach using MI is the joint MI (JMI),^57^ which focuses on the complementary information between features toward estimating the response, which for a candidate feature $f_{j}$ is

$$\text{JMI}\overset{\text{def}}{=}\underset{j\;\in \;Q-S}{\text{max}}\sum_{s\;\in \;S}I\left(f_{j},f_{s};y\right).$$

The underlying concept in JMI is to include candidate features that are complementary with already existing features in the feature subset S since, intrinsically, it computes the pairwise information of features taken jointly with the response.

The double input symmetrical relevance (DISR)^58^ is a modification of the JMI criterion by normalizing MI using the joint entropy:

$$\text{DISR}\overset{\text{def}}{=}\underset{j\;\in \;Q-S}{\text{max}}\sum_{s\;\in \;S}\frac{I\left(f_{j},f_{s};y\right)}{H\left(f_{j},f_{s},y\right)}.$$

The conditional MI maximization criterion (CMIM)^59^ is another approach that relies on MI to select a candidate feature conditioning upon the features that have already been selected in the feature subset:

$$\text{CMIM}\overset{\text{def}}{=}\underset{j\;\in \;Q-S}{\text{max}}\left\{\min_{s\;\in \;S}\left[I\left(f_{j};y|f_{s}\right)\right]\right\}.$$

The conditional infomax feature extraction (CIFE)^60^ explicitly attempts to integrate all three key concepts in FS, relevance, redundancy, and complementarity and takes the form

$$\text{CIFE}\overset{\text{def}}{=}\underset{j\;\in \;Q-S}{\text{max}}\left[I\left(f_{j},y\right)-\sum_{s\;\in \;S}I\left(f_{j},f_{s}\right)+\sum_{s\;\in \;S}I\left(f_{j},f_{s}|y\right)\right].$$

The L1-squared-loss MI (L1-LSMI)^9^ has been conceptually developed to integrate feature interaction and L1-regularization to maximize the squared-loss variant of MI between selected features and the response. This builds on the premise that estimating the density ratio may be a more efficient approach toward assessing variable dependencies compared with estimating the marginal and joint densities (or similarly entropies), which are computationally challenging for the accurate estimation of MI.^61^

The quadratic programming feature selection (QPFS)^62^ expresses the FS task as a quadratic-programming problem attempting to provide a global solution compared with the greedy approaches summarized above. Conceptually, QPFS attempts to make a global decision considering the interaction across all features jointly; however, Vinh et al.^26^ have highlighted some challenges with the QPFS framework including tackling problems with small number of samples or potentially resultant non-convex formulations in empirical experiments.

The SPECCMI algorithm^26^ has a similar conceptual grounding to QPFS aiming for a global MI-based FS solution. They proposed a spectral relaxation for efficiently solving a quadratic-integer-programming problem, which jointly considers relevance, unconditional redundancy, and class-conditional redundancy.

#### Implementations of FS algorithms

The performance details of the FS algorithms may differ using different implementations, so it is important to also highlight the source code used. For GSO, we used Guyon’s implementation, which can be found in the appendix of that study.^23^ For mRMR, we used Peng’s implementation (https://uk.mathworks.com/matlabcentral/fileexchange/14608-mrmr-feature-selection-using-mutual-information-computation) and for mRMR Spearman the implementation by Tsanas (https://github.com/ThanasisTsanas/mRMR_Spearman). We remark that Peng’s code discretizes continuous features to efficiently compute MI (as most implementations computing MI do). For information gain, CFS, and CBF, we used the WEKA implementations (https://www.cs.waikato.ac.nz/ml/weka/) through a MATLAB interface from the ASU FS repository. The ASU FS repository provides a range of FS algorithms and was originally implemented in MATLAB (https://jundongl.github.io/scikit-feature/OLD/home_old.html) and, more recently, in Python (https://jundongl.github.io/scikit-feature/). For the purposes of this study, we used the MATLAB implementation. For JMI and DISR, we used the fast implementation in FEAST (https://github.com/Craigacp/FEAST).^13^ This implementation requires the discretization of continuous features for the computation of MI and following the authors' experimental setup that was set to 5 levels. Similarly, for QPFS, MIQ, CMIM, CIFE, and SPECMI, we used the implementation of Vinh (https://uk.mathworks.com/matlabcentral/fileexchange/47129-information-theoretic-feature-selection), who also recommended 5 levels of discretization. For LOGO, we used the MATLAB implementation by the developer of the algorithm (https://www.acsu.buffalo.edu/∼yijunsun/lab/LOGO.html). For IAMB and HITON, we used the MATLAB implementation in the Causal Explorer by the developers of the algorithms (https://github.com/mensxmachina/CausalExplorer_1.5), although we need to note that this is not open-source code (in the sense that the functions are executable but the implementations are not visible to developers). For L1-LSMI, we used the code by the developer of the package (Jitkrittum) (https://github.com/wittawatj/l1lsmi). For RELIEF, we used MATLAB’s native implementation (command “relief”, https://uk.mathworks.com/help/stats/relieff.html).

#### Novel FS algorithm: RRCT

We propose a new principled FS heuristic algorithm, RRCT, which attempts to effectively integrate all three major components outlined above for effective FS (relevance, redundancy, and complementarity) in a computationally efficient scheme. The proposed CBF FS algorithm extends the mRMR Spearman concept discussed in the preceding section by adjusting the original relevance and redundancy terms and incorporating a complementarity term. It relies on the computation of correlation coefficients, which are subsequently transformed using a function inspired by IT concepts. We invoke these IT concepts under the assumption that the features are normally distributed, which is common in diverse machine-learning applications and often works well in practice.^63^ This assumption greatly facilitates analysis since important IT concepts that are of central importance to this new algorithm are simple to compute and to work with analytically.

The first step is to standardize features to have zero mean and unit standard deviation before further processing to ensure there is no feature clearly dominating others due to the measurement scales. Subsequently, we compute the Spearman rank correlation coefficient between the features and the response to obtain the vector of rank correlations $\;r=\left[r_{1},r_{2}\ldots r_{M}\right]$, where each entry denotes the correlation of each feature with the response. We used the Spearman rank correlation coefficient over the linear correlation coefficient as a more general method to express the relationship between variables. Then, we compute the covariance matrix, Σ, and denote its entries, $\rho_{\text{ij}}$: these entries are the Spearman rank correlation coefficients computed between the features $f_{i}$ and $f_{j}$, where i,jε(1…M).

(Equation 1) $$\Sigma =\left[\begin{array}{llll} 1 & \rho_{12} & \ldots & \rho_{1M}\\ \rho_{12} & 1 & \ldots & \rho_{2M}\\ \vdots & \vdots & \ddots & \vdots \\ \rho_{1M} & \rho_{2M} & \ldots & 1 \end{array}\right]$$

For the Gaussian distribution, there is an analytic expression for MI that depends only on the linear correlation coefficient ρ^27^ (strictly speaking, MI also relies on the variance, but this is 1 due to the standardization step):

(Equation 2) $$MI=-0.5\cdot \ln\left(1-\rho^{2}\right).$$

Equation 2 leads to an IT quantity (MI) that is obtained using the linear correlation coefficient. Here, we will use the same notion to define an IT quantity exactly as in Equation 2, except this time the Spearman correlation coefficient will be used. For convenience, we will use the notation $r_{\text{IT}}\left(X,Y\right)=-0.5\cdot \text{log}\left[1-r_{XY}^{2}\right]$ to refer to the non-linearly transformed rank correlation coefficient $r_{XY}$ between two random variables, X,Y. Now, we can write in compact vector form all the relevance terms using the IT-inspired transform in Equation 3:

(Equation 3) $$r_{\text{ITL}}=-0.5\cdot \log\left[1-\begin{array}{lll} r_{1}^{2} & \cdots & 1-r_{M}^{2} \end{array}\right].$$

Similarly, using the covariance matrix Σ and Equation 3, the redundancy between pairs of features can be conveniently expressed as a matrix, where each (i,j) entry denotes the information that two features share in predicting the response:

(Equation 4) $$\Sigma_{\text{IT}}=-0.5\cdot \log\left[\begin{array}{llll} 1 & 1-\rho_{12}^{2} & \ldots & 1-\rho_{1Μ}^{2}\\ 1-\rho_{12}^{2} & 1 & \cdots & 1-\rho_{2Μ}^{2}\\ \vdots & \vdots & \ddots & \vdots \\ 1-\rho_{1Μ}^{2} & 1-\rho_{2Μ}^{2} & \ldots & 1 \end{array}\right].$$

Now, inserting the relevance terms in Equation 3 across the main diagonal of $\Sigma_{\text{IT}}$ in Equation 4, we obtain a matrix that will be used to compute the compromise between relevance and redundancy:

(Equation 5) $$D=-0.5\cdot \log\left[\begin{array}{llll} 1-r_{1}^{2} & 1-\rho_{12}^{2} & \ldots & 1-\rho_{1Μ}^{2}\\ 1-\rho_{12}^{2} & 1-r_{2}^{2} & \cdots & 1-\rho_{2Μ}^{2}\\ \vdots & \vdots & \ddots & \vdots \\ 1-\rho_{1Μ}^{2} & 1-\rho_{2Μ}^{2} & \ldots & 1-r_{M}^{2} \end{array}\right].$$

The matrix D is essentially a compact form of mRMR, which alleviates the need for repeated computation of the relevance and complementarity terms in the iterative steps (therefore, this expedites the incremental FS process in large datasets). Conceptually, the IT transformation of the rank correlation coefficient assigns greater weight to coefficients above the absolute value 0.5 (see Figure 5). The effect is that weak statistical associations (between a feature and the response or between features) are penalized; conversely, strong associations (large absolute correlation coefficients) are enhanced. If the absolute value of the rank correlation coefficient is 1, we set the MI quantity to a very large value (we chose 1,000).

The proposed algorithm developed thus far can be seen as a computationally simpler version of the classical mRMR, which can be computationally efficiently computed because the information has been succinctly summarized in matrix **D**, so that for the computation of the new candidate feature $f_{j}$ (which corresponds to a feature not in the existing feature subset), we focus on the i^th^ row. The relevance of the feature $f_{j}$ lies on the main diagonal of the matrix **D**, and the redundancy is computed from the average of the terms that appear in the column s (the D_*i*,*s*_ entries), where s corresponds to features in the already selected subset S (which contains the indices s of the selected features).

The following step is crucial for the development of RRCT: we embrace the concept of quantifying the conditional relevance (complementarity) of a feature as the usefulness of that feature in predicting the response conditional upon the already selected feature subset. This is achieved using the rank partial correlation coefficient, which quantifies the statistical association between two random variables, X,Y, while controlling for the effect of a set of a conditioning random variable, Z. This is defined as

(Equation 6) $$r_{\text{p}}\left(X,Y|Z\right)=\frac{N\cdot \sum_{i=1}^{N}r_{X,i}\cdot r_{Y,i}-\sum_{i=1}^{N}r_{X,i}\cdot \sum_{i=1}^{N}r_{Y,i}}{\sqrt{N\cdot \sum_{i=1}^{N}r_{X,i}^{2}-{\left(\sum_{i=1}^{N}r_{X,i}\right)}^{2}}\cdot \sqrt{N\cdot \sum_{i=1}^{N}r_{Y,i}^{2}-{\left(\sum_{i=1}^{N}r_{Y,i}\right)}^{2}}},$$

where $r_{X,i}$ and $r_{Y,i}$ denote the residuals of X,Y, respectively, on Z. That is, the partial correlation coefficient is computed by first solving the two associated linear-regression problems and calculating the correlation between their residuals. Alternatively, the partial correlation coefficient can be computed using a recursive formula working directly with correlation coefficients: the n^*th*^-order partial correlation (that is, the conditioning random variable Z contains n features) is computed from three (n−1)-order partial correlations (the 0^th^-order partial correlations are, by definition, the correlation coefficients). For the simplest case where the conditioning random variable Z comprises a single feature, this reduces to Equation 7:

(Equation 7) $$r_{\text{p}}\left(X,Y|Z\right)=\frac{r\left(X,Y\right)-r\left(X,Z\right)\cdot r\left(Y,Z\right)}{\sqrt{r^{2}\left(X,Z\right)}\cdot \sqrt{r^{2}\left(Y,Z\right)}}.$$

The partial correlation coefficient expresses the contribution of the independent random variable X over and above the contributions of the conditioning random variable Z for predicting the dependent random variable Y and accounts for the additional explanation of the variance observed in Y as a result of including X in the regression setting. Figure 6 presents a Venn diagram to graphically illustrate this point where the different regions denote the information captured by each random variable, and the overlapping regions denote the shared information between the random variables.

In the context of the developed FS algorithm, the partial correlation coefficient $r_{\text{p}}$ is defined as the rank correlation coefficient between a new candidate feature, $f_{j}$, and the response y, controlling for the existing features in the subset, i.e., $r_{\text{p}}\left(f_{j},y|S\right)$. This approach aims to incorporate how well the candidate feature pairs up with the existing features that have already been chosen. Then, we transform the computed partial correlation coefficient using the IT-inspired transformation in Equation 2, which gives

(Equation 8) $$r_{\text{p},\;\text{IT}}=-0.5\cdot \log\left[1-r_{p}^{2}\right].$$

Since the controlling variables S (whose effect needs to be removed to compute the partial correlation coefficient) are not known and will vary at each step, it is not possible to express this quantity in compact vector or matrix form as we did previously for **D**. The term in Equation 8 is thus computed separately in each step, giving rise to the final equation that is used toward selecting features in RRCT:

(Equation 9) $$\begin{array}{l} \text{RRCT}\overset{\text{def}}{=}\max_{j\;\in \;Q-S}[\underset{relevance}{\underset{︸}{r_{\text{IT}}\left(f_{j},y\right)}}-\underset{redundancy}{\underset{︸}{\frac{1}{\left|S\right|}\sum_{s\;\in \;S}r_{\text{IT}}\left(f_{j},f_{s}\right)}}\\ \;+\underset{complementarity}{\underset{︸}{\text{sign}\left(r_{\text{p}}\left(f_{j},y|S\right)\right)\cdot \text{sign}\left(r_{\text{p}}\left(f_{j},y|S\right)-r\left(f_{j},y\right)\right)\cdot r_{\text{p},\;\text{IT}}}}]. \end{array}$$

sign(⋅) returns +1 if the quantity (⋅) is positive and −1 if (⋅) is negative and is used to determine whether $r_{\text{p},\text{ IT}}$ is added or subtracted in Equation 9.

Care needs to be exercised in the RRCT expression when including the $r_{\text{p},\text{ IT}}$ term. Given that this term is non-negative due to the IT transformation, we need to determine whether the inclusion of the candidate feature to the existing subset actually contributes additional information conditional on the features in the selected subset (conditionally relevant). Consideration must be made for both the sign of the partial correlation coefficient and for the sign of the difference in magnitudes between $r_{\text{p}}\left(f_{j},y|S\right)$ and $r\left(f_{j},y\right)$. The $\text{sign}\left(r_{\text{p}}\left(f_{j},y|S\right)-r\left(f_{j},y\right)\right)$ term in Equation 9 is used to determine whether the conditional relevance $r_{\text{p}}\left(f_{j},y|S\right)$ is larger than $r\left(f_{j},y\right)$ magnitude; that would suggest that including the candidate feature has additional (conditional) relevance given the features in the selected subset. The $\text{sign}\left(r_{\text{p}}\left(f_{j},y|S\right)\right)$ term is used to make the overall complementarity contribution positive in the case that $r\left(f_{j},y\right)<0$, $r_{\text{p}}\left(f_{j},y|S\right)<0$ and $\left(r_{\text{p}}\left(f_{j},y|S\right)-r\left(f_{j},y\right)\right)<0$ because then the term $\text{sign}\left(r_{\text{p}}\left(f_{j},y|S\right)-r\left(f_{j},y\right)\right)$ would indicate that the additional contribution offered by the complementarity term is negative.

RRCT uses Equation 9 to incrementally select a feature and place it in the selected set of features S. Specifically,

RRCT computation

1. Select and place the first feature with index j: $\underset{j\;\in \;Q}{\text{max}}\left(r_{\text{IT}}\left(f_{j},y\right)\right)$ in the initially empty set S, that is {j}→S. Q contains the indices of all the features in the initial *M*-dimensional feature space.
2. Selecting the next m−1 features, one at each step, by repeating the following: apply the criterion in Equation 9 to select the next feature index j not already selected (i.e., from the Q−S set) and include it in the set S∪{j}→S. The relevance and redundancy terms are conveniently pre-computed and directly used from matrix D (see Equation 5).
3. Obtain the feature subset by selecting the features ${\left\{f_{j}\right\}}_{j=1}^{m}$, jεS from the original data matrix X.

This reduced feature subset is the new data matrix $X^{\left(m\right)}\;\varepsilon \;R^{N\times m}$, which can be used for further processing.

#### FS strategy

Each of the FS algorithms used herein provide a ranked output of the selected feature subset in descending order, where the first selected feature is deemed to be the most predictive of the response (where each FS algorithm has their own internal criteria for achieving this) and progressively working toward the successively less predictive features of the response. Optionally, some of the investigated algorithms also provide feature weights.

When we apply FS algorithms to determine a feature subset to assess whether the true set of features have been selected (e.g., in synthetic datasets), we can proceed with using the entire data matrix X. However, when selecting a feature subset aiming to assess performance and when limited to a single dataset for selecting features and assessing model generalization performance, we need to be more careful. This is because we risk potentially biasing findings if we use the entire data matrix to select the feature subset and only subsequently proceed with standard model validation methods such as CV. Instead, feature sets need to be selected using a training set and evaluated on the testing set, for example, using CV, which is the standard model validation approach that has been typically used in FS literature for determining the feature set.

Ideally, we should obtain the same feature subset across all CV replications; this would clearly indicate what features should be selected in the dataset for a given FS algorithm. However, in practice, the selected features for any given FS algorithm may be different across different CV replicates. Hence, we need to develop a strategy to determine the selected features and the order with which they appear in the selected feature subset for each FS algorithm. Specifically, we follow the methodology we have previously described,^15^^,^^30^^,^^31^ which is summarized in Table 5. This methodology is generic and can be readily applied with any greedy FS algorithm, i.e., all those FS algorithms that select features one at a time (moreover, it can be extended to alternative, non-greedy FS algorithms, see Tsanas^15^).

### FS assessment

This section describes the methodology for the assessment of the FS algorithms when applied to the datasets summarized in Table 4.

There are two approaches to evaluate the performance of FS algorithms: (1) assessing whether the “optimal” feature subset was selected, i.e., identifying the true features contributing to estimating the response and discarding probes and redundant features, and (2) presenting the selected feature subsets into a subsequent supervised-learning algorithm (classifier or regressor, depending on the problem) and using a pre-defined performance measure for comparison. The former is only possible in synthetic datasets, where the true features are known in advance. The latter is a surrogate approach to evaluate the performance of FS algorithms^64^ by introducing an additional layer into the FS problem and does not necessarily correspond to selecting the true feature subset. In practice, some weakly relevant or redundant features could improve the learners' performance; conversely, the benefit of discarding relevant features may outweigh loss in information content.^22^ Moreover, it is possible that using different learners might lead to different conclusions regarding the superiority of the FS algorithms.^20^ In practice, both approaches are commonly used in FS literature^3^^,^^7^^,^^64^ and will be used in this study as well to empirically compare the performance of RRCT against established widely used filter FS algorithms.

#### FS assessment using synthetic data

In synthetic datasets, the optimal feature subset is known *a priori*, and therefore we can evaluate whether the FS algorithms identify the true feature subset. Given that the number of true and false (collectively referring to redundant, irrelevant, and noisy) features in a synthetic dataset is known, we can progressively assess how well FS algorithms identify the true features. Specifically, we used the FDR, defined as the ratio of the number of false features identified by the FS algorithm as belonging to the selected feature subset over the total (true and false) number of selected features (i.e., number of false features estimated out of three features, out of four features, etc.) at a given step. The FDR lies in the range [0 … 1], where 0 indicates correctly identifying all features and 1 indicates selecting only false features (probes). Ideally, FDR should remain at zero for as many steps as the true features in the dataset, and we can use a plot to identify at what step FS algorithms select false features.

For the synthetic data, all samples were used to determine the final feature subset.

#### FS assessment using real-world data

To conform with the research literature on evaluating FS algorithms, we employ a statistical learner into which we present the selected features from the different FS algorithms. Given that we use this statistical-learning step to infer the performance of the FS algorithms, we wanted to use a statistical learner that would provide good performance, making full use of potential feature interactions, and not require any further tuning so that it provides a fair assessment of the FS step. For these reasons, we opted to use a RF herein because it is a powerful statistical learner that is very robust to the tuning of its hyper-parameters.^1^^,^^65^ We used the default RF parameters: 500 trees, growing all trees fully, searching for the best split of the data by accessing in the tree nodes only the square root of the number of features (randomly selected), and using majority voting for the final RF estimate. This is because ultimately we did not aim to maximize performance but rather to objectively infer FS performance on the basis of the statistical learner’s outputs.

As mentioned previously, each FS algorithm used in the study provides the selected feature subset in descending order of preference, and here we presented the 1 … *K* selected features from each FS algorithm into the RF, where we set *K* to be the smallest number between 30 and the dataset dimensionality. In each step, we recorded the misclassification rate (number of samples assigned into the wrong class over the total number of samples presented into RF) and, for convenience, expressed that rate as a percentage. Ideally, we want this misclassification percentage score to be zero and can infer that an FS algorithm outperforms competing approaches when exhibiting a lower score.

For both the FS and the model validation in the real-world datasets, we used the following rules:

(1) If separate training and testing subsets were provided (see Table 4), then we used the training data to select features and train the model then evaluated performance on the testing data.
(2) If no separate training and testing subsets were provided, we used 10-fold CV to select features and assess performance when having more than 150 samples; otherwise, we used a leave-one-sample-out approach to select features and evaluate model performance.

### Time-complexity analysis

A key practical consideration when choosing algorithms in general (and for the purposes of this study, FS algorithms in particular) includes understanding their time complexity, i.e., the running time. The running time depends on the computer configuration (computer and compiler or software) used, and therefore that needs to be reported along with the running time of specific algorithms across the examined practical tasks.^66^

It is also possible to express the time complexity of an algorithm using the “big-oh” notation, which aims to represent the time complexity, e.g., as a function of the inputs into the algorithm.^66^ However, as Aho and Ullman have noted, “quite often, the running time of a program depends on a particular input, not just on the size of the input,” in which case we need to consider the worst-case running time.^66^ Therefore, when time complexity is considered in FS literature, researchers typically report the actual running time of algorithms along with the used computer configuration. We similarly provide this empirical comparison of FS algorithms in this study, reporting their running time across each of the 8 real-world datasets.

## Data Availability

All datasets used in the study are freely and publicly available. Most are available from the UCI ML Repository (https://archive.ics.uci.edu/ml/index.php). Additionally, the ovarian-cancer dataset is freely available from http://www.biomedcentral.com/1471-2105/10/259/additional, and the SCRBCT dataset is available from http://www-stat.stanford.edu/∼tibs/ElemStatLearn/. The Relathe dataset is freely available from https://jundongl.github.io/scikit-feature/datasets.html. We have provided specific links to where each of the datasets can be downloaded when describing the data (see Table 4). The synthetic data were generated by adjusting publicly available data generators and are available at https://github.com/ThanasisTsanas/RRCT/tree/main/Data. For convenience and easier reference, all synthetic and real-world datasets used in this study are included in that Github link in MATLAB (∗.mat) file format and Excel (∗.xlsx) file format, the latter being a more generic and accessible filetype that can be used across programming languages. The MATLAB code developed and used in this study is freely available on the author’s group website under https://www.darth-group.com/software and the author’s Github project page (https://github.com/ThanasisTsanas/RRCT). Furthermore, RRCT has been implemented in Python (currently slower than the MATLAB version) and is available at the aforementioned Github link. The intention is to keep the code in the Github link up to date; for the frozen version, which was used to generate the results in this study using the MATLAB code, see the Zenodo (https://doi.org/10.5281/zenodo.6139462).

## Supplemental information

Document S1. MATLAB source code for the RRCT algorithm

Document S2. Article plus supplemental information
