# Supplementary material for: Relevance, redundancy, and complementarity trade-off (RRCT): A principled, generic, robust feature-selection tool
Source: Patterns (N Y). 2022 Mar 31;3(5):100471. doi: 10.1016/j.patter.2022.100471 (PMC9122960; doi:10.1016/j.patter.2022.100471)
Supplement: Document S1. MATLAB source code for the RRCT algorithm [file mmc1.pdf]

Patterns, Volume *3*

Supplemental information

Relevance, redundancy, and complementarity trade-off (RRCT): A principled, generic, robust feature-selection tool

Athanasios Tsanas

```
function [features, RRCT_all] = RRCT(X, y, K)
%
% General call: [features] = RRCT(X, y);
%               [features, RRCT_all] = RRCT(X, y, 30);
%
%% Function for feature selection (FS), based on the RRCT concept
RELEVANCE, REDUNDANCY AND COMPLEMENTARITY TRADE-OFF (RRCT)
%
% This function is based on solid theoretical principles, integrating the
% key concepts for FS about feature relevance, redundancy, and conditional
% relevance (complementarity)
%
% Aim: select the best features out of a matrix NxM
%
% Inputs:  X       -> N by M matrix, N = observations, M = features
%          y       -> N by 1 vector with the numerical outputs
%__________________________________________________________________________
% optional inputs:
%          K       -> number of features to be selected (integer>0)
[default = M, i.e. the dimensionality of the dataset, OR K=30, whichever is lower]
%
% =========================================================================
% Output:
%        features  -> Selected feature subset in descending order
%        RRCT_all  -> All the outputs in a struct: relevance, redundancy,
%                     complementarity and FS algorithm's output
% =========================================================================
%
% Part of the "Tsanas FSToolbox"
%
% -----------------------------------------------------------------------
% Useful references:
%
% 1) A. Tsanas: "Relevance, redundancy and complementarity trade-off (RRCT):
%    a generic, efficient, robust feature selection tool", Patterns,
%    (in press), 2022
% 2) H. Peng, F. Long, and C. Ding: "Feature selection based on mutual
%    information: criteria of max-dependency, max-relevance, and
%    min-redundancy", IEEE Transactions on Pattern Analysis and Machine
%    Intelligence, Vol. 27, No. 8, pp.1226-1238, 2005
% 3) R. Battiti: "Using mutual information for selecting features in
%    supervised neural net learning", IEEE Transactions on Neural Networks,
%    Vol. 5(4), pp. 537–550, 1994
% 4) A. Tsanas: "Accurate telemonitoring of Parkinson's disease symptom
%    severity using nonlinear speech signal processing and statistical
%    machine learning", D.Phil. thesis, University of Oxford, UK, 2012
% -----------------------------------------------------------------------
%
% Modification history
% --------------------
% 18 Feb 2022: Making code available along with published manuscript
%
% -----------------------------------------------------------------------
% (c) Athanasios Tsanas
%
```

```
% ********************************************************************
% If you use this program please cite:
%
% 1) A. Tsanas: Relevance, redundancy and complementarity trade-off (RRCT):
%    a generic, efficient, robust feature selection tool, Patterns,
%    (in press), 2022
% ********************************************************************
%
% For any question, to report bugs, or just to say this was useful, email
% tsanasthanasis@gmail.com
%
% *** For updates please check: https://github.com/ThanasisTsanas/RRCT ***
%

%% Set defaults
% Determine rows N and columns M of the input matrix X
[N,M] = size(X);

if nargin<3  || isempty(K)
    K = min(M, 30);
end

if (K>M) % guard against the case the user has indicated more features than the
dimensionality of the dataset
    K=M; disp('You provided K>M, i.e. more features to be ranked than the
dimensionality of the data, reverting to K=M!')
end

% help RRCT
% disp('press any key'); pause

tStart = tic;

%% Data preprocessing

% Guard against missing values (discard these points)
j = all(~isnan([X y]),2); X = X(j,:); y = y(j);

% Pre-process the input matrix X to compute the z-scores in the columns
X = zscore(X);

% Make y a column vector if it isn't already
y=y(:);

%% Compute relevance, redundancy and build the mRMR matrix

relv = corr(X, y, 'type', 'Spearman', 'rows', 'pairwise');
j = isnan(relv); relv(j)=0;

% **** RELEVANCE BY DEFINITION
relevance = -0.5*log_bb(1-relv.^2, 'Nats', Inf);
clear j; j = isnan(relevance); relevance(j) = 0;
clear j; j = isinf(relevance); relevance(j) = 1000;

redundancy = corr(X, 'type', 'Spearman', 'rows', 'pairwise');
```

```
redundancy = redundancy - diag(ones(1,M));

% **** REDUNDANCY BY DEFINITION
redundancy = -0.5*log_bb(1-redundancy.^2, 'Nats', Inf);
clear j2; j2 = isinf(redundancy); redundancy(j2) = 1000;

% Define convenient matrix
mRMR_matrix = redundancy;
mRMR_matrix = mRMR_matrix + diag(relevance);

%% Main loop to obtain the feature subset

RRCT_metric = NaN(1,K);
[rel_val, idx1] = sort(relevance,'descend');
features(1) = idx1(1);
RRCT_metric(1) = rel_val(1);

% initialize vectors that will hold the relevance, redundancy and complementarity
values
RRCT_all.relevance(1) = rel_val(1);
RRCT_all.redundancy(1) = 0;
RRCT_all.complementarity(1) = 0;

for k = 2:K
    candidates = 1:M; candidates(features) = []; % potential candidates at this step

    mean_redundancy = mean(mRMR_matrix(candidates, features),2); % compute average
redundancy for the kth step
    clear fs_idx complementarity;
    complementarity = partialcorr(X(:,candidates), y, X(:,features), 'type',
'Spearman', 'rows', 'complete');
    Csign = sign(complementarity).*sign(complementarity-relv(candidates));
    complementarity = -0.5*log_bb(1-(complementarity).^2, 'Nats', Inf);
    clear j3; j3 = isinf(complementarity); complementarity(j3) = 1000;

    % RRCT HEART: Max relevance - min redundancy + complementarity optimization
    [RRCT_metric(k), fs_idx] = max(relevance(candidates) - mean_redundancy +
Csign.*complementarity);
    features(k) = candidates(fs_idx);

    % store the three elements: Relevance, Redundancy, Complementarity
    RRCT_all.relevance(k) = relevance(features(k));
    RRCT_all.redundancy(k) = mean_redundancy(fs_idx);
    RRCT_all.complementarity(k) = Csign(fs_idx)*complementarity(fs_idx);
end

RRCT_all.RRCT_metric = RRCT_metric;

tEnd = toc(tStart);
disp(['Total Execution Time: ', num2str(tEnd), ' seconds.']);

end % end of main function
```

```
%% ========================================================================
%               Additional functions for the RRCT program

function pout = log_bb(pin, method, pin_val)

% Function that computes the algorithm depending on the user specified
% base; if the input probability is zero it returns the pin_val (by default this is
0).

% Utility function
% A. Tsanas, August 2010

%% Check inputs and set defaults

if nargin<3 || isempty(pin_val)
    pin_val = 0; % default substituting value in case "pin" is zero
end

if nargin<2 || isempty(method)
    method = 'Nats';
end

switch (method)
    case 'Hartson' % using log10 for the entropy computation
        log_b=@log10;

    case 'Nats' % using ln (natural log) for the entropy computation
        log_b=@log;

    otherwise % method -> 'Bits' using log2 for the entropy computation
        log_b=@log2;
end

%% Actual log computation using the log base determined above

pout = log_b(pin); % default computation using the method selected above; then in the
next step take out the cases where we had samples being equal to zero (which would
make the entropy become NaN)
pout(pin==0) = pin_val; % correct for the cases where we have log(0), set it to the
pin_val (typically zero)

end % end of the log_bb function
```
